# Two‐Dimensional Infrared Spectroscopy Reveals the Presence of a Bridging CO Ligand in Two Catalytic Intermediates of [FeFe] Hydrogenase

**DOI:** 10.1002/anie.2628759

**Published:** 2026-04-10

**Authors:** Cornelius C. M. Bernitzky, Mathesh Vaithiyanathan, Manon T. Lachmann, Igor V. Sazanovich, Gregory M. Greetham, Patricia Rodríguez‐Maciá, James A. Birrell, Marius Horch

**Affiliations:** ^1^ Department of Physics, Ultrafast Dynamics in Catalysis Freie Universität Berlin Berlin Germany; ^2^ School of Chemistry and Leicester Institute for Structural and Chemical Biology University of Leicester Leicester UK; ^3^ STFC Central Laser Facility, Research Complex at Harwell, Rutherford Appleton Laboratory Harwell Campus Didcot UK; ^4^ School of Life Sciences University of Essex Colchester UK

**Keywords:** catalytic mechanism, green hydrogen, metalloenzyme, vibrational spectroscopy, organometallic

## Abstract

[FeFe] hydrogenases are highly active, reversible enzymes for the interconversion of hydrogen with protons and electrons. Their active site H‐cluster consists of a canonical [4Fe‐4S] cluster covalently linked to a unique [2Fe]_H_ centre. Their catalytic mechanism has been studied extensively, but several details remain disputed, and two rival models exist in the literature. One crucial difference between these models is the structure and catalytic relevance of two states named H_red_H^+^ and H_sred_H^+^. In the first model, these states are catalytic intermediates containing a reduced [Fe(I)Fe(I)]_H_ centre and a bridging CO ligand (µCO), while in the second model they are inactive states containing an oxidised [Fe(II)Fe(II)]_H_ site and a bridging hydride ligand (µH^−^). The second proposal was initially based on the lack of a prominent absorption peak attributed to a µCO ligand in the infrared (IR) spectra of both states. Here, we provide evidence for the presence of a µCO ligand in the H_red_H^+^ and H_sred_H^+^ states using two‐dimensional (2D) IR spectroscopy, firmly establishing the structure of these states as [Fe(I)Fe(I)]_H_ with a µCO ligand. The results suggest that these states are catalytically relevant intermediates with crucial implications for understanding hydrogen conversion in nature and designing new synthetic catalysts.

## Graphical abstract

Two‐dimensional (2D) infrared (IR) spectroscopy provides vastly more structural and functional insight into chemical and biological systems than linear IR spectroscopy. Here, we apply 2D‐IR spectroscopy to two controversially discussed states of the [FeFe] hydrogenase active site, revealing the presence of a bridging carbonyl ligand, which is difficult to observe in linear IR spectra, supporting the notion that these states are crucial catalytic intermediates.

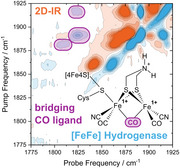

## 1 Introduction

Hydrogenases are metalloenzymes that catalyse the reversible production of dihydrogen (H_2_) from protons and electrons [1, 2, 3, 4, 5, 6, 7, 8, 9]. Of the three classes of hydrogenases ([Fe], [FeFe], [NiFe]), the [FeFe] hydrogenases are reported to be most active and reversible, catalysing H_2_ production at rates up to 10 000 s^−1^ and H_2_ oxidation rates of over 100 000 s^−1^ [10, 11]. Understanding their catalytic mechanism may enable the design of new synthetic catalysts with comparable activities based on earth‐abundant elements.

**FIGURE 1:**
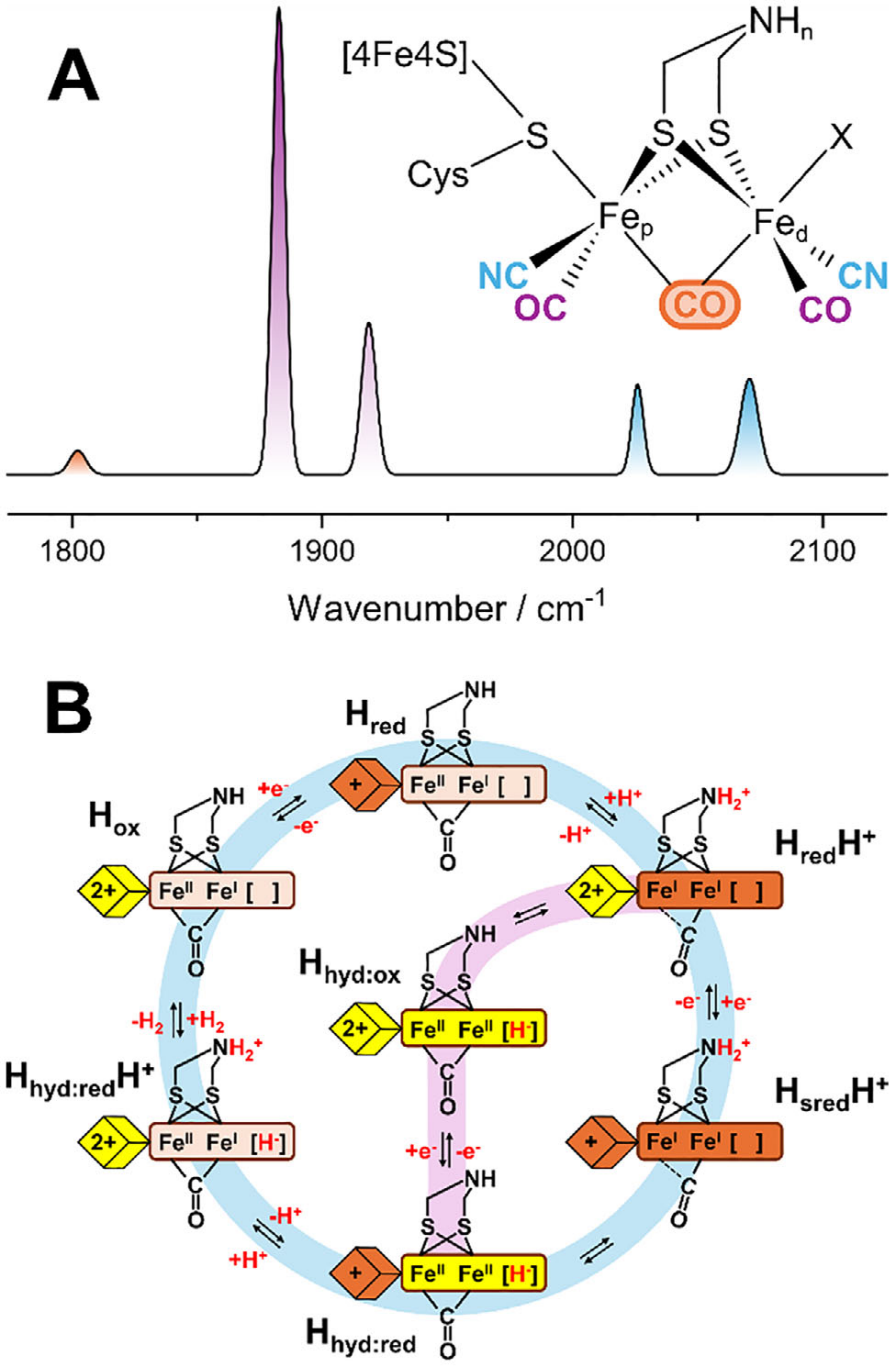
(A) Structure of the active site H‐cluster of [FeFe] hydrogenase alongside an idealised FTIR spectrum of the H_sred_H^+^ state. “*n*” indicates the possibility that the bridgehead nitrogen can accommodate 1 or 2 H^+^. (B) Proposed catalytic cycle in which H_red_H^+^ and H_sred_H^+^ are integral catalytic intermediates.

The [FeFe] hydrogenase's active site H‐cluster (Figure 1A) is composed of a canonical [4Fe‐4S] cluster ([4Fe‐4S]_H_) covalently linked through a cysteine thiolate to a unique diiron site ([2Fe]_H_) [12, 13]. [2Fe]_H_ is the site of hydrogen formation and oxidation, while [4Fe‐4S]_H_ acts as an electron storage site. The two Fe ions of [2Fe]_H_, the proximal (Fe_p_) and distal (Fe_d_) Fe, named based on their distance from [4Fe‐4S]_H_, are coordinated by one terminal CO and CN^−^ ligand each and bridged by two thiolates of a unique 2‐azapropane‐1,3‐dithiolate (ADT) ligand as well as an additional CO (for all structurally characterised states) [14, 15]. The CO and CN^−^ ligands yield five structurally sensitive and spectrally isolated bond‐stretching vibrations that can help identify different states of the H‐cluster by infrared (IR) spectroscopy.

Several heatedly debated mechanistic proposals have been formulated [1]. In one model (Figure 1B), the oxidised active state, H_ox_, consisting of an oxidised [4Fe‐4S]_H_ ([4Fe‐4S]_H_^2+^) and a mixed valence [2Fe]_H_ ([Fe_p_(II)Fe_d_(I)]_H_), is reduced during H_2_ production by adding one electron to [4Fe‐4S]_H_, generating the H_red_ state ([4Fe‐4S]^+^‐[Fe_p_(II)Fe_d_(I)]) [16, 17]. It is debated whether this process is coupled to protonation of [4Fe‐4S]_H_ (there is also nomenclature discrepancy) [18, 19]. H_red_ is then thought to protonate on the bridgehead nitrogen of the ADT ligand, triggering electron transfer from [4Fe‐4S]_H_ to [2Fe]_H_, yielding a [4Fe‐4S]_H_^2+^‐[Fe_p_(I)Fe_d_(I)]H^+^ configuration in the H_red_H^+^ state [20, 21]. This frees up [4Fe‐4S]_H_ for a second reduction, generating the H_sred_H^+^ state with a [4Fe‐4S]_H_^+^‐[Fe_p_(I)Fe_d_(I)]H^+^ configuration [22]. Both H_red_H^+^ and H_sred_H^+^ are reported to be in tautomeric equilibrium with terminal hydride‐bound states called H_hyd:ox_ and H_hyd:red_, respectively, with [4Fe‐4S]_H_^2+^‐[Fe_p_(II)Fe_d_(II)]H^−^ and [4Fe‐4S]_H_^+^‐[Fe_p_(II)Fe_d_(II)]H^−^ configurations [23]. H_hyd:red_ is just one proton away from forming H_2_, and it is hypothesised to protonate at the ADT ligand, followed by electron transfer from [4Fe‐4S]_H_ to [2Fe]_H_, proton transfer to the terminal hydride forming H_2_, and finally H_2_ release [23].

The structure and catalytic importance of H_red_H^+^ and H_sred_H^+^ remain controversial, especially for extensively studied high‐activity model enzymes from *Chlamydomonas reinhardtii* (*Cr*HydA1) and *Desulfovibrio desulfuricans* (*Dd*HydAB). While we and others have proposed the [Fe(I)Fe(I)]H^+^ configuration with a bridging CO ligand (µCO) [1, 9, 23, 24, 25], others have argued that these states are tautomers in which the proton oxidises the Fe ions in [2Fe]_H_, forming a bridging hydride (µH^−^) state, [4Fe‐4S]_H_^2+^‐[Fe_p_(II)Fe_d_(II)]µH^−^, as an off‐pathway low‐activity species (Figure S1) [7, 18, 26, 27]. A central piece of evidence for this µH^−^ structure is the perceived lack of a µCO peak in the IR spectra of H_red_H^+^ and H_sred_H^+^ and the appearance of an additional peak in the terminal CO region [16, 17, 22]. However, some [FeFe] hydrogenases, specifically sensory enzymes, feature a well‐detectable µCO signal in the IR spectra of H_red_H^+^ and H_sred_H^+^ [25, 28]. The activity of these enzymes is low, though, so that statements about the catalytic involvement of their (super‐)reduced states are difficult to make. However, low‐temperature IR studies also revealed the appearance of a µCO peak in H_red_H^+^ and H_sred_H^+^ states of *Cr*HydA1 and *Dd*HydAB [23, 24, 25], suggesting that the additional *terminal* CO band is attributable to H_hyd:ox_ and H_hyd:red_ [20]. However, the relevance of these low‐temperature studies has been challenged, and the forms of H_red_H^+^ and H_sred_H^+^ observed at room temperature and low temperature were suggested to be different states [27]. We instead argue that the µCO ligand is difficult to detect at room temperature for most [FeFe] hydrogenases due to peak broadening and an intrinsically low and temperature‐dependent extinction coefficient (*vide infra*).

To distinguish between these two scenarios (µCO vs. no µCO) and provide further insight into the structure of these two controversial catalytic intermediates, we decided to use two‐dimensional (2D) IR spectroscopy. Briefly, 2D‐IR spectroscopy is a nonlinear technique utilising a sequence of ultrashort IR pulses to access multiple vibrational energy levels of a molecule. In the pump‐probe implementation, two pump pulses with a controlled time delay typically generate a population in vibrationally excited states, while a probe pulse measures the resulting change in absorbance after a certain waiting time, *T*_W_. Using this approach, detailed insights into the potential energy surface of a molecule, as well as structural and vibrational dynamics, are obtained, as recently demonstrated for both [NiFe] and [FeFe] hydrogenases [29, 30, 31, 33]. In particular, 2D‐IR spectroscopy reveals anharmonic coupling of different vibrational modes. In this way, peaks observed in the linear IR absorption spectrum can unambiguously be assigned to specific states through cross‐peaks in the 2D‐IR spectrum. In addition, the nonlinear nature of the experiment facilitates the identification of weak transitions, as associated cross‐peaks may gain intensity from coupled bright modes.

## 2 Results and Discussion

**FIGURE 2:**
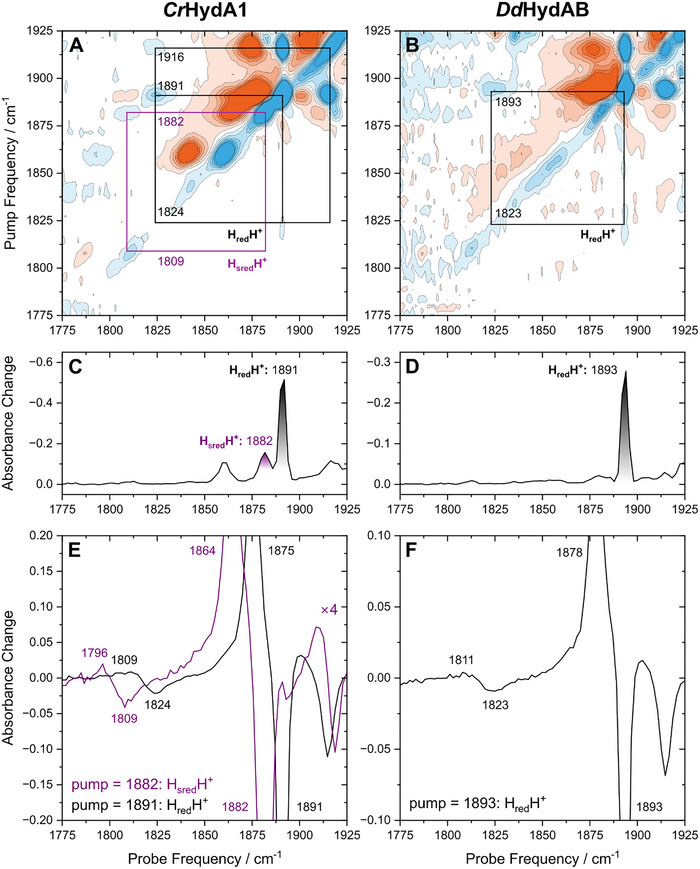
2D‐IR spectra of (A) *Cr*HydA1 and (B) *Dd*HydAB, reduced with sodium dithionite at pH 8. The corresponding sign‐inverted diagonal spectra, similar to linear IR spectra, are shown in panels (C) and (D), respectively. Cross‐peaks between the most intense tCO mode and the µCO mode are highlighted for H_red_H^+^ (black) and H_sred_H^+^ (purple) by pump slices through the 2D‐IR spectra of (E) *Cr*HydA1 and (F) *Dd*HydAB. Spectra were recorded with perpendicular pump‐probe polarisation at *T* = 283 K and *T*_W_ = 0.25 ps.

The 2D‐IR spectra of high‐activity [FeFe] model hydrogenases from *Chlamydomonas reinhardtii* (*Cr*HydA1) and *Desulfovibrio desulfuricans* (*Dd*HydAB) reduced with sodium dithionite, recorded with perpendicular polarisation of pump and probe pulses at *T* = 283 K and *T*_W_ = 0.25 ps, are shown in Figure 2A,B, respectively (spectra recorded with parallel polarization did not yield any extra information). The negative (blue) features on the diagonal (extracted in Figure 2C,D) represent the fundamental transitions of individual vibrational modes that are also seen in the linear IR spectra, while the positive (orange) peaks immediately to the left of them correspond to transitions from an excited state (*v* = 1, *v* = 2, etc.) to higher excited states (*v* = 2, *v* = 3, etc.). Off‐diagonal cross‐peaks indicate coupling between pairs of vibrational modes. Each of these cross‐peaks has a negative (blue) and a positive (red) part. If two modes are coupled, pumping one of them will bleach the shared vibrational ground state. As a consequence, absorption by the second mode is decreased, and a negative signal at the fundamental transition energy is observed, giving a negative cross‐peak. In addition, the same pump process generates a population in the vibrationally excited state of the first mode. The second mode can now be excited from this state, yielding a positive cross‐peak, typically at energies slightly lower than the fundamental transition. The latter feature may not be well‐observable, though, in some cases, for example, due to a small transition dipole moment of the sequence transition [29, 30, 33]. Diagonal and off‐diagonal features are observed for the CO and CN stretching modes of all contributing states, leading to complex signatures in the 2D‐IR spectra (see Figures S2–S10 for a detailed assignment). However, both H_red_H^+^ and H_sred_H^+^ can be clearly identified by their most prominent diagonal signals, which are associated with one of the terminal CO (tCO) stretching modes. These features are observed for H_red_H^+^ at 1891 cm^−1^ (*Cr*HydA1) and 1893 cm^−1^ (*Dd*HydAB) and for H_sred_H^+^ at 1882 cm^−1^ (*Cr*HydA1). The H_sred_H^+^ state is not clearly observed for *Dd*HydAB in these experiments, as the redox potential for its formation is far more negative than for *Cr*HydA1 [16, 32]. Pumping these bright transitions yields cross‐peaks between these most intense peaks of H_sred_H^+^ and H_red_H^+^ and most of the other peaks attributed to them. In addition, clearly observable cross‐peaks with bleach contributions at pump/probe frequencies of 1891/1824 cm^−1^ and 1882/1809 cm^−1^ are observed for *Cr*HydA1 and 1893/1823 cm^−1^ for *Dd*HydAB, indicating the presence of a coupled µCO vibration at 1824 cm^−1^ (*Cr*HydA1), 1823 cm^−1^ (*Dd*HydAB) for H_red_H^+^, and 1809 cm^−1^ (*Cr*HydA1) for H_sred_H^+^. Of note, no prominent diagonal features in the 1800–1830 cm^−1^ region can be detected for H_red_H^+^ or H_sred_H^+^. While minor contributions of H_sred_H^+^ to a diagonal signal observed for *Cr*HydA1 at 1809/1811 cm^−1^ cannot be excluded, this feature mainly corresponded to another state, H_ox_‐CO (see Figure S5), and no such diagonal signal is observed for the H_red_H^+^ states in *Cr*HydA1 or *Dd*HydAB, *vide infra*. This finding highlights the difficulty of directly probing these vibrations, as previously observed in linear IR absorption experiments [24]. However, the observation of the identified coupling cross‐peaks at early waiting times unambiguously reveals the presence of these vibrations and their affiliation with the assigned H_red_H^+^ and H_sred_H^+^ states, thereby providing evidence for the presence of a µCO ligand in both states at ambient temperature. Of note, the observed cross‐peaks are much more intense than the diagonal transitions since two of the three incoming light‐matter interactions (those associated with the pump beam) involve the pronounced transition dipole moment of the bright tCO stretch modes (rather than the weak transition dipole moment of the µCO stretch mode). In addition, the linewidths of the involved transitions do not exceed the off‐diagonal anharmonicity. Therefore, positive and negative contributions to the cross‐peak do not cancel, in contrast to many other systems where this effect leads to a lowered cross‐peak intensity. For *Cr*HydA1, there are also weak cross‐peaks at 1808/1885 cm^−1^ and 1822/1891 cm^−1^, further supporting the assignment of the µCO mode of the H_red_H^+^ state. Importantly, no cross‐peaks are observed between the most intense peaks identifying H_red_H^+^ (1891 and 1893 cm^−1^) and H_sred_H^+^ (1882 cm^−1^) to any diagonal feature in the 1950–1990 cm^−1^ region (Figure S2). This observation indicates that the peaks observed in this range are not attributed to the H_sred_H^+^ and H_red_H^+^ states, agreeing with their previous assignment to the tautomeric forms H_hyd:red_ and H_hyd:ox_ (*vide infra*) [23]. Regardless of their origin, we can conclude that no peaks in the 1950–1990 cm^−1^ region are associated with H_red_H^+^ and H_sred_H^+^.

The features observed in the 2D plots can also be highlighted by taking horizontal “slices” at specific pump frequencies. For *Cr*HydA1, slices at pump frequencies of 1883 cm^−1^ (H_sred_H^+^) and 1891 cm^−1^ (H_red_H^+^) reveal bleach signals in the µCO region at 1809 and 1824 cm^−1^, respectively (Figure 2E). Likewise, pumping at 1805 and 1822 cm^−1^ reveals bleach signals at 1882 and 1891 cm^−1^ (Figures S3 and S4). Notably, bleaching is much more pronounced if the bright terminal CO stretch mode is pumped. If these bright modes at 1882 cm^−1^ and 1891 cm^−1^ are pumped, it is also possible to observe bleaches at other frequencies associated with fundamental transitions of H_sred_H^+^ and H_red_H^+^, respectively (Figures S3 and S4), further supporting the identity of the probed states.

Importantly, no obvious features in the 1950–1990 cm^−1^ region are observed in slices at pump frequencies corresponding to known peaks from H_sred_H^+^ or H_red_H^+^ neither for *Cr*HydA1 nor *Dd*HydAB (Figures S3, S4, and S10). This further confirms unambiguously that, in contrast to previous suggestions [27], H_sred_H^+^ of *Cr*HydA1 and H_red_H^+^ of *Cr*HydA1 and *Dd*HydAB do not feature additional terminal CO ligands at ambient temperatures. This finding is fully consistent with the observation of a µCO mode in these states at both ambient and cryogenic conditions [24], that is, H_red_H^+^ and H_sred_H^+^ have the same structure at all investigated temperatures. The unambiguous observation of a µCO ligand negates the possibility of these states containing a µH^−^ ligand. This implies minimal structural reorganisation during the formation of these states. We conclude, therefore, that H_red_H^+^ and H_sred_H^+^ are catalytic intermediates that form part of the main catalytic cycle for producing and oxidising hydrogen. They both contain a fully reduced diiron site that is capable of tautomerising to terminal hydride‐containing states, H_hyd:ox_ and H_hyd:red_, which also suggests protonation of the amine bridgehead [23]. So far, H_hyd:ox_ and H_hyd:red_ have only been substantially populated under cryogenic illumination of H_red_H^+^ and H_sred_H^+^ [23]. However, the previous observation of IR peaks in the 1950–1990 cm^−1^ region may indicate the presence of a proportion of these states at room temperature [23, 24]. We attempted to quantify the proportion of the hydride‐tautomers in room‐temperature linear IR spectra (Figure S11). We also investigated the influence of buffer composition, pH and salt concentration and found little effect (Figures S12 and S13; Table S2).

**FIGURE 3:**
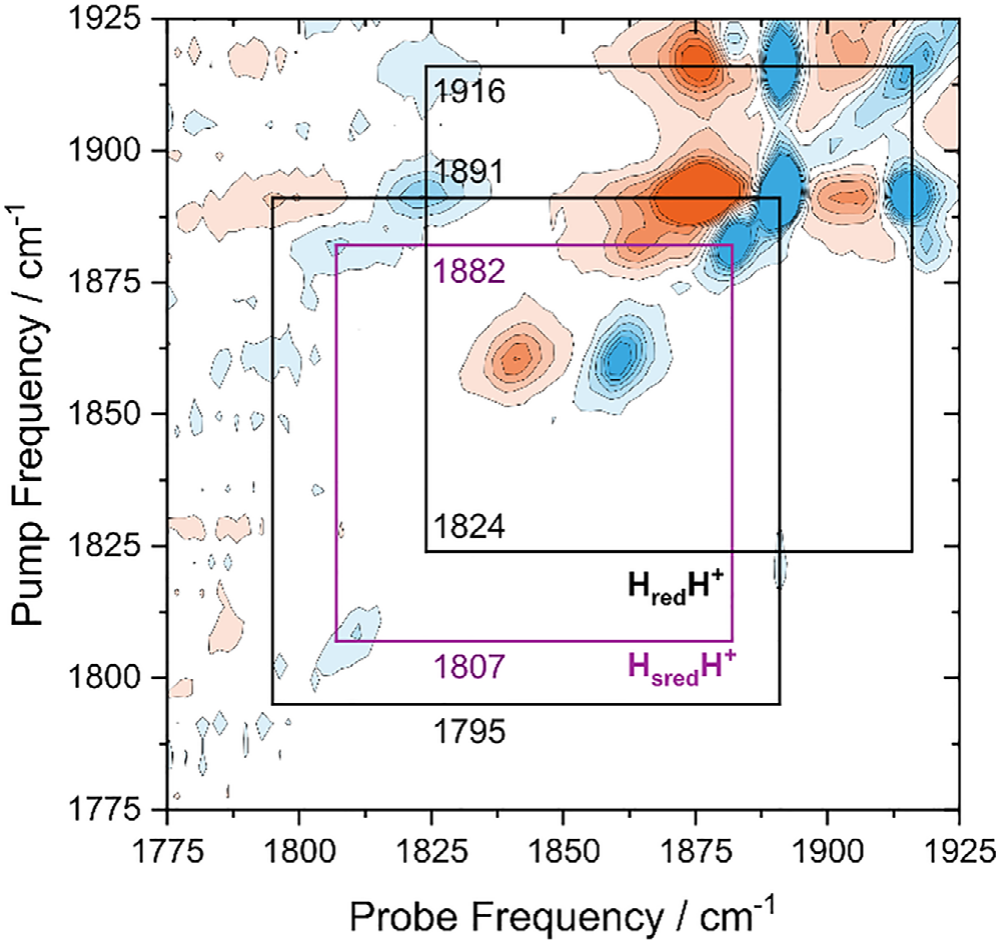
2D‐IR spectrum of *Cr*HydA1, reduced with sodium dithionite at pH 8. The spectrum was recorded with perpendicular pump‐probe polarisation at *T* = 283 K and *T*_W_ = 3 ps. Another dataset with a higher population of H_sred_H^+^ is shown in Figure S14. Note that the frequency of the positive cross‐peak of H_red_H^+^ (1795 cm^−1^) is likely underestimated, due to strong broadening (see Figure S14). Likewise, the negative cross peak of H_sred_H+ (1807 cm^−1^) is lower in frequency than observed at *T*_W_ = 0.25 ps (1809 cm^−1^, see Figure 2A,E). The value obtained at *T*_W_ = 3 ps may be more reliable (see Figure S14), but, for the sake of consistency, we refer to the value initially obtained at *T*_W_ = 0.25 ps throughout the manuscript.

The 2D‐IR cross peaks discussed above result from anharmonic coupling between tCO modes and the µCO mode. Therefore, they are observed from the earliest waiting times on, providing unambiguous proof that both types of modes correspond to the same state, that is, H_red_H^+^ and H_sred_H^+^. Apart from anharmonic coupling, cross peaks in 2D‐IR spectra can also arise from energy transfer between vibrational modes. In that case, the cross peaks grow into the spectrum as a function of the waiting time, *T*_W_. Notably, the separation of positive and negative parts in energy‐transfer cross peaks does *not* reflect the *off‐diagonal* anharmonicity, i.e. the coupling between the two modes. Instead, the separation reflects the *diagonal* anharmonicity of the mode that has *not* been pumped, reflecting population transfer from the pumped mode. As a consequence, cross peaks from energy transfer provide additional insights into dark modes that do not produce noticeable diagonal signals. Focusing on data from *Cr*HydA1 and the H_red_H^+^ state, which showed higher overall signal intensities (*vide supra*), we therefore recorded 2D‐IR spectra at a longer waiting time of *T*_W_ = 3 ps (Figure 3). Pumping the brightest tCO mode of H_red_H^+^, clear cross peaks can be identified that link the most prominent tCO signal at 1891 cm^−1^ to a dark transition at 1824 cm^−1^. Consistent with the above findings from early waiting times (*T*_W_ = 0.25 ps), we assign this dark transition to the µCO mode of H_red_H^+^. Further proof for this assignment comes from inspecting the separation between positive and negative parts of the cross peaks. While the coupling cross peaks discussed above show a separation of 10–15 cm^−1^ (Figure 2E), the separation in the energy‐transfer cross peak is significantly larger, on the order of ≥ 25 cm^−1^. This value reflects the diagonal anharmonicity of the dark transitions at 1824 cm^−1^, which can be extracted since its first vibrationally excited state has been populated via energy transfer from the pumped bright mode. The obtained value matches general expectations for a bond‐localized stretching vibration of a metal‐bound CO ligand [29, 30, 33, 34, 35, 36, 37, 38] and, more specifically, the value observed for the µCO mode of [FeFe] hydrogenase [31]. Similar features can also be observed for H_sred_H^+^ in individual datasets (Figure S14), but the signals are typically less pronounced due to a lower population of this intermediate. In total, the dark transition at 1824 and 1809 cm^−1^reflect µCO modes, as evident from observed diagonal and off‐diagonal anharmonicities (Figure S15) as well as low fundamental frequencies, thereby proving the presence of a bridging CO ligand in both H_red_H^+^ and H_sred_H^+^.

**FIGURE 4:**
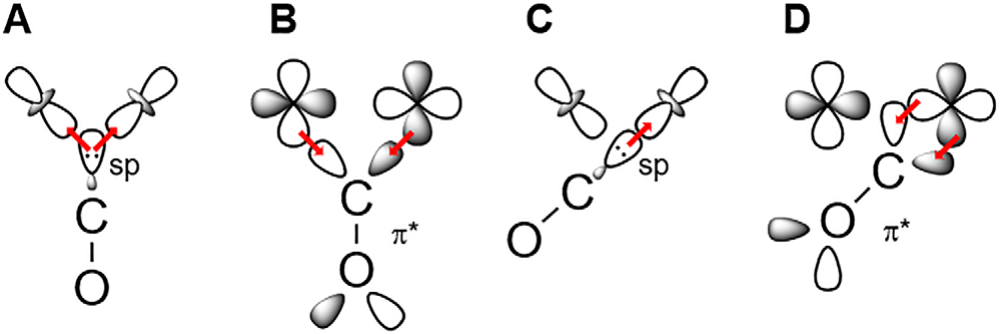
Scheme showing the metal‐ligand binding in bridging (A, B) and terminal (C, D) CO arrangements. The filled CO σ molecular orbital donates electron density into the (partly) empty metal e_g_ *d*‐orbitals (*d*_x_^2^_−y_^2^ or *d*_z_^2^, A and C). The filled metal t_2g_ d‐orbitals (*d*_xy_, *d*_xz_, or *d*_yz_) donate electron density into the empty CO π* molecular orbital. The orientation of the CO ligand that is favoured will depend on the orbital overlap and occupancy of the metal e_g_ *d*‐orbitals. In the low‐spin Fe(II) state, both e_g_ orbitals are empty; in the low‐spin Fe(I) state, there is one electron in one of the e_g_ orbitals, and the other is empty.

An interesting observation is that the µCO mode in H_red_H^+^ and H_sred_H^+^ appears at a higher frequency at room temperature (1824 and 1809 cm^−1^) than at cryogenic temperatures (1817 and 1801 cm^−1^) [23, 24]. The 7–8 cm^−1^ difference may be due to differences in the vibrationally averaged structure (varying degrees of thermally excited low‐frequency modes), which seems to yield a more bridging configuration (with low µCO frequency) at cryogenic temperatures and a semi‐bridging one (with higher µCO frequency) at room temperature. A plot of peak position vs. temperature for the µCO mode in H_sred_H^+^ of *Cr*HydA1 shows a linear trend between 40 and 200 K (Figure S16). It is also interesting that the µCO modes in H_red_H^+^ and H_sred_H^+^, in which [2Fe]_H_ has acquired an extra reducing equivalent, are not downshifted with respect to the µCO peak observed in states with an Fe(II)Fe(I) ground state, that is, H_ox_ (1803 cm^−1^ for *Cr*HydA1 [21]; 1802 cm^−1^ for *Dd*HydAB [32]) and H_red_ (1791 cm^−1^ for *Cr*HydA1 [21]; 1792 cm^−1^ for *Dd*HydAB [32]). Meanwhile, the main terminal CO ligands are downshifted by about 50 cm^−1^ (1883/1891 cm^−1^ in H_sred_H^+^/H_red_H^+^ vs. 1933/1939 cm^−1^ in H_red_/H_ox_ for *Cr*HydA1 [21] and 1883/1893 cm^−1^ in H_sred_H^+^/H_red_H^+^ vs. 1934/1940 cm^−1^ in H_red_/H_ox_ for *Dd*HydA1 [32]). This suggests that the µCO ligand becomes less bridging and more terminal in H_sred_H^+^ and H_red_H^+^ compared with H_ox_/H_red_, which counteracts the red shift caused by adding an additional electron onto the [2Fe]_H_ site (Figure 4). Additional electron density on the Fe ions is thought to be donated into the π* antibonding molecular orbitals of the CO (and CN^−^) ligands. If the bridging CO partially shifts toward Fe_d_ in the H_red_H^+^ and H_sred_H^+^ states, due to significant population of the d_z_^2^ orbital and the pronounced *trans* influence of the cysteine on Fe_p_, there will be reduced π backdonation from Fe_p_, which will compensate for the increased π backdonation due to the extra electron on [2Fe]_H_.

## 3 Conclusion

In conclusion, we discount that H_red_H^+^ and H_sred_H^+^ are bridging‐hydride‐containing off‐pathway states (assumed to be low activity). Instead, these states were identified as CO‐bridged intermediates that form a critical part of the catalytic cycle, allowing the H_ox_ resting state to be transformed to tautomeric hydride states with minimal structural reorganisation, thereby facilitating H_2_ evolution at unrivalled rates. The central involvement of these reduced states also implies a mechanism where protonation of the ADT bridgehead atom and electron accumulation on the [2Fe]_H_ center are intimately coupled. In particular, the more reduced H_hyd:red_ hydride state, a tautomer of H_sred_H^+^, is likely to become protonated on the bridgehead ADT nitrogen, yielding a H_hyd:red_H^+^‐type state. We speculate that this step involves electron transfer from [4Fe‐4S]_H_ to [2Fe]_H_, yielding an H_ox_‐like electronic ground state. Such a state would have a [2Fe]_H_ cluster that is too electron‐rich, thereby weakening the Fe–H^−^ bond and favouring protonation of the hydride to yield H_2_. Subsequent studies using further ultrafast IR techniques coupled to isotope‐exchange studies on protonation and hydride‐formation steps could provide evidence for this proposed mechanism, specifically the catalytic competence of the H_red_H^+^ and H_sred_H^+^ states, and may yield additional insight into the catalytic cycle of [FeFe] hydrogenases.

## Conflicts of Interest

The authors declare no conflicts of interest.

## Supporting information



The authors have cited additional references within the Supporting Information [39, 40, 41, 42, 43, 44, 45, 46].

## Data Availability

The data that supports the findings of this study are available from the corresponding author upon reasonable request.
