# Supplementary material for: Two‐Dimensional Infrared Spectroscopy Reveals the Presence of a Bridging CO Ligand in Two Catalytic Intermediates of [FeFe] Hydrogenase
Source: Angew Chem Int Ed Engl. 2026 Apr 10;65(21):e2628759. doi: 10.1002/anie.2628759 (PMC13182199; doi:10.1002/anie.2628759)
Supplement: Supplementary file 1 — The authors have cited additional references within the Supporting Information [39, 40, 41, 42, 43, 44, 45, 46]. [file ANIE-65-e2628759-s001.pdf]

Supplementary Information

Two-Dimensional Infrared Spectroscopy Reveals the Presence of a Bridging CO Ligand in Two Catalytic Intermediates of [FeFe] Hydrogenase

Cornelius C. M. Bernitzky[a], Mathesh Vaithiyanathan[a], Manon T. Lachmann[b], Igor V. Sazanovich,[c] Gregory M. Greetham,[c] Patricia Rodriguez Macia[b], James A. Birrell[d*], Marius Horch[a*]

[a] C. C. M. Bernitzky, M. Vaithiyanathan, Dr. M. Horch
Department of Physics, Ultrafast Dynamics in Catalysis
Freie Universität Berlin
Arnimallee 14, 14195 Berlin, Germany
E-mail: marius.horch@fu-berlin.de

[b] Manon T. Lachmann, Dr. P. Rodríguez-Maciá
School of Chemistry and Leicester Institute for Structural and Chemical Biology
University of Leicester
Leicester, LE1 7RH, UK

[c] Dr. I. V. Sazanovich, Dr. G. M. Greetham
STFC Central Laser Facility, Research Complex at Harwell,
Rutherford Appleton Laboratory, Harwell Campus
Didcot, OX11 0QX, UK

[d] Dr. J. A. Birrell
School of Life Sciences
University of Essex
Wivenhoe Park, Colchester, CO4 3SQ, UK
E-mail: james.birrell@essex.ac.uk

Contents

Experimental Details p. S1

Supplementary Figures S1–S16 p. S2

Supplementary Tables S1–S2 p. S18

Supplementary References p. S19

Experimental details

*Cr*HydA1 and *Dd*HydAB (ca. 2 mM) were prepared as described previously.[1-2] Samples, in 100 mM Tris-HCl, 150 mM NaCl, pH 8, were mixed with 50 mM sodium dithionite in an anaerobic glovebox under an Ar atmosphere and transferred to gas-tight IR cells. Samples were shielded from light and oxygen as much as possible to minimise formation of the H_{ox}-CO state by damage to the active site and release of free CO.

2D-IR spectra were recorded in transmission mode using a gas-tight and temperature-controlled (T = 283 K) small-volume sandwich cell (optical path length = 50 µm, $V \approx 8$ µL) equipped with CaF_2 windows. All data were acquired in pump-probe geometry utilizing mid-IR pulses (centre frequency = 1950 cm^{-1}; bandwidth > 300 cm^{-1}; pulse duration = 50 fs; repetition rate = 10 kHz; pump energy: 1 µJ; probe energy: 100 nJ) from the ULTRA laser-system as described previously.[3-6] 2D-IR data (accumulation time = 300 s) were obtained with perpendicular pump-probe polarization at a waiting time of T_W = 250 fs. The 2D-IR data were obtained in a time-domain fashion by scanning the coherence time τ between two pulse-shaper generated collinear pump pulses from 0 to 6 ps (step size 30 fs) prior to overlap with the probe pulse and self-heterodyned detection of the collinearly emitted signal.[3-5] The pump frequency axis was obtained by Fourier transformation of the time-domain signal with respect to τ (spectral resolution ≈ 2.8 cm^{-1}), while the probe frequency axis was obtained by signal dispersion in two spectrographs and detection *via* two liquid-nitrogen cooled 128-element MCT detectors (spectral resolution < 2.5 cm^{-1}). Four-frame phase cycling was employed in 2D-IR data acquisition to limit contributions from pump light scattered on the detector.[4]

Supplementary Figures

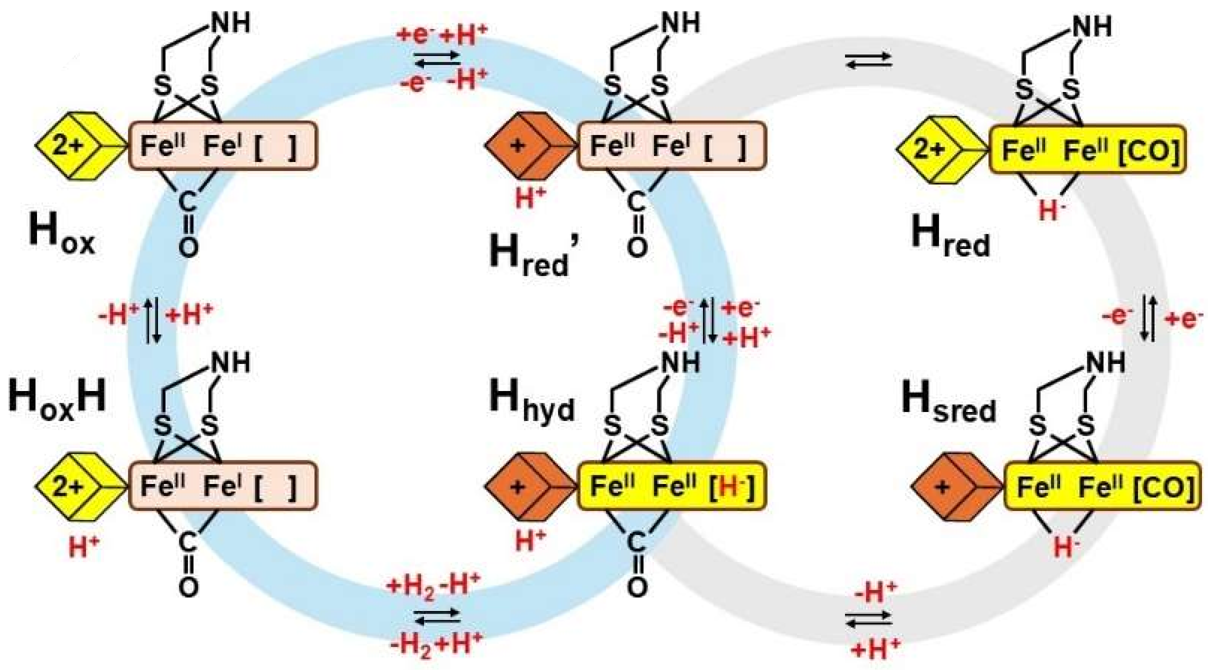

Figure S1: Alternative proposal for the catalytic cycle in which $H_{red}H^+$ and $H_{sred}H^+$ are considered to be off-pathway. The structures proposed for these and, thus, the derived model for the catalytic cycle are in conflict with the results of the current study.

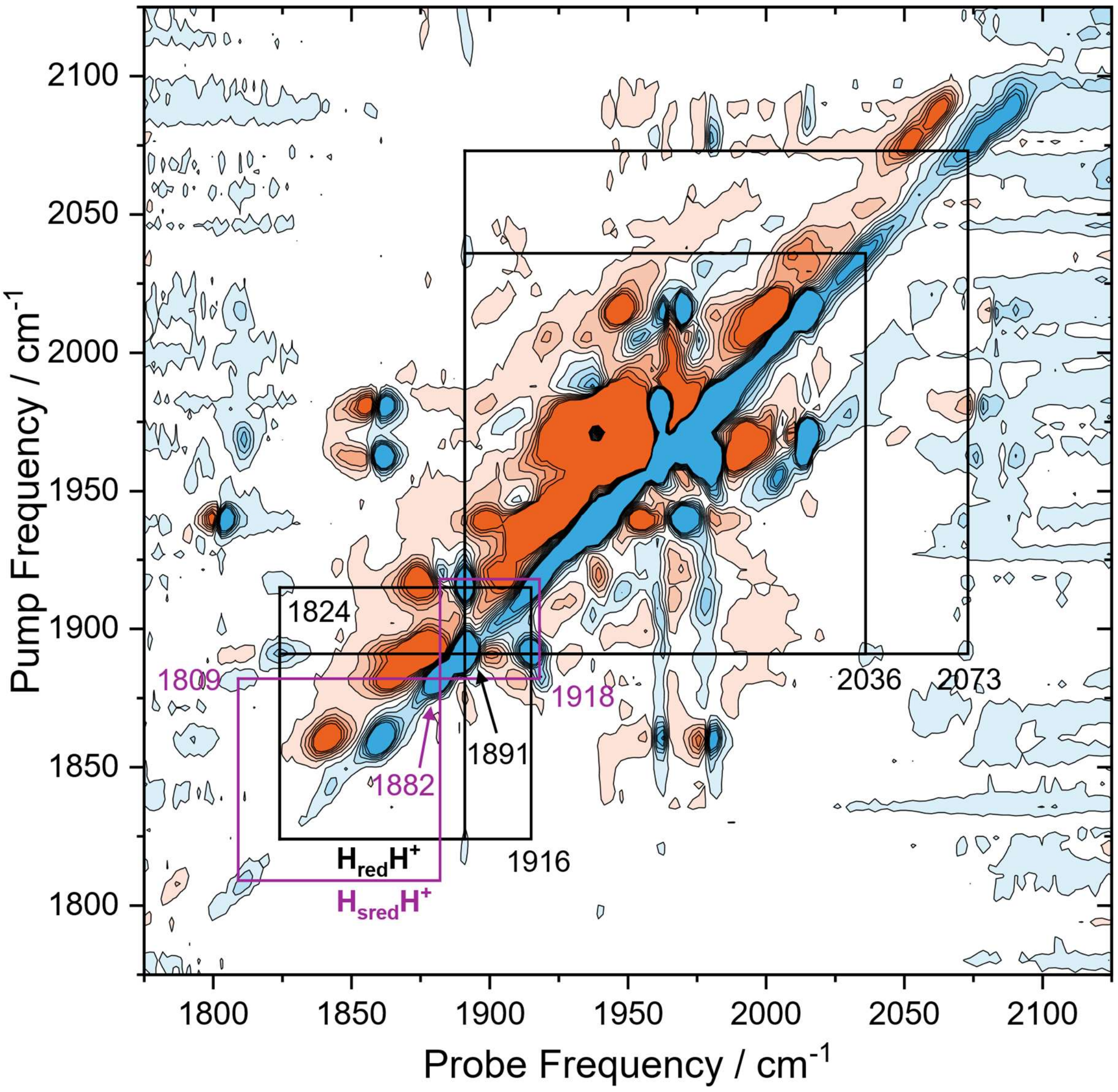

Figure S2: Full-range 2D-IR spectrum of reduced *Cr*HydA1 (Fig. 2A), covering all CO and CN stretch-mode signals. Prominent signals related to $H_{red}H^+$ and $H_{sred}H^+$ are highlighted in black and purple, respectively. The spectrum was recorded with perpendicular pump-probe polarization at a waiting time of T_W = 250 fs at T = 283 K.

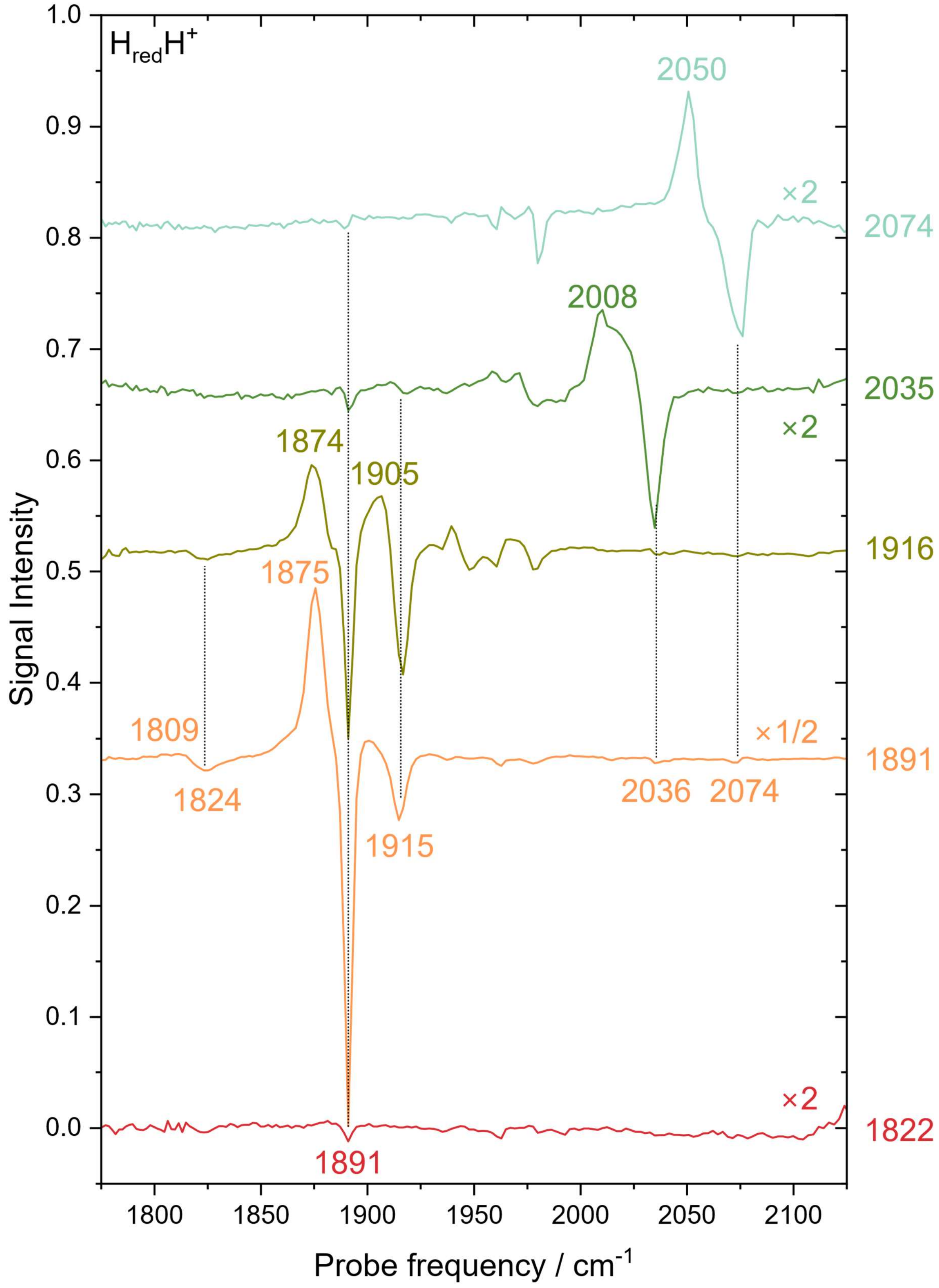

Figure S3: Pump slices through the full range 2D-IR spectrum of reduced *Cr*HydA1 as shown in Fig. S2. The pump frequencies correspond to the fundamental transitions of the $H_{red}H^+$ state. The pump slice at 1916 cm^{-1} has H_{ox} contributions and very weak bleach signals indicating coupling to several CN modes, including those of the $H_{red}H^+$ state, expected at 2036 and 2074 cm^{-1}.

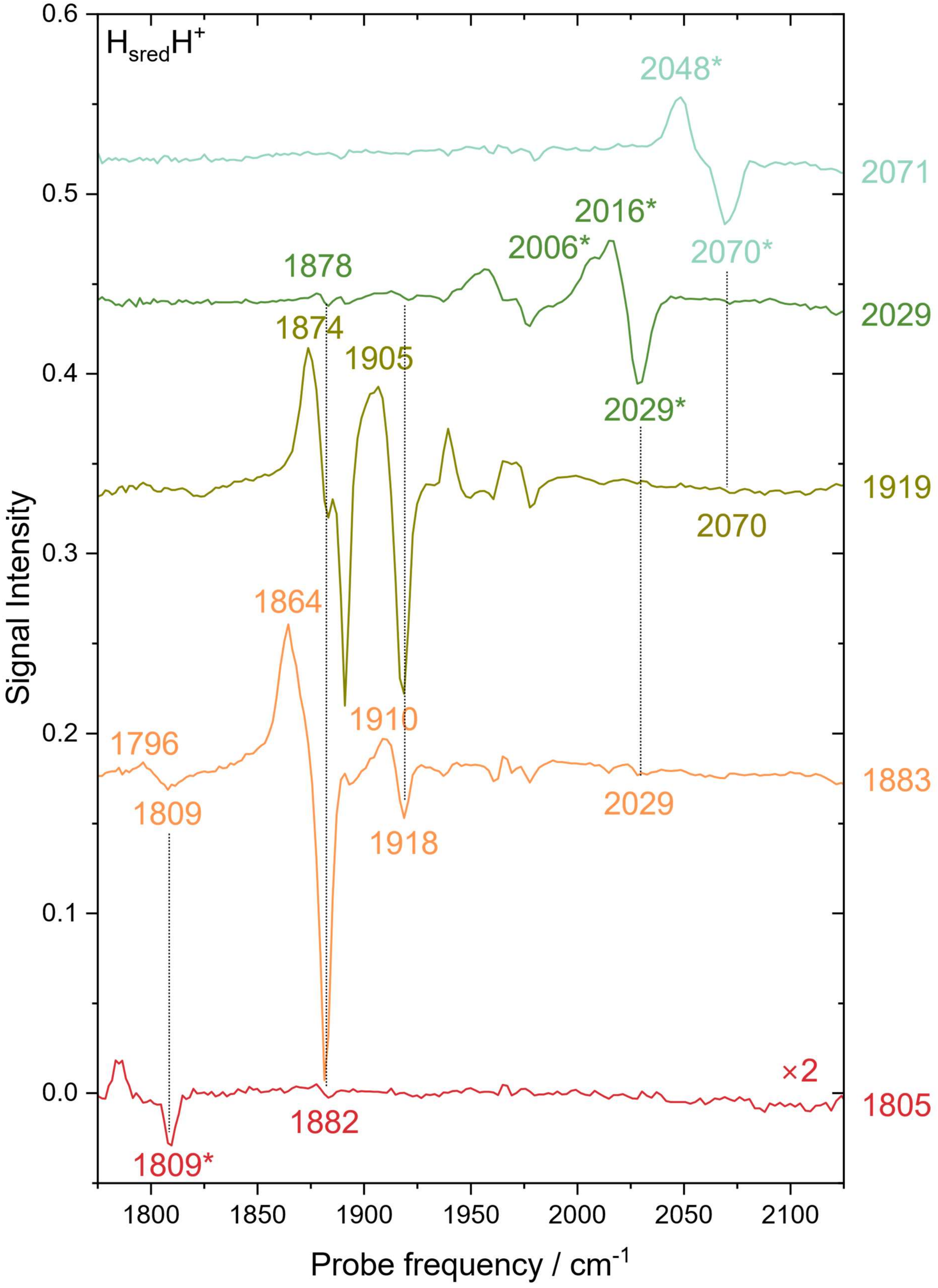

Figure S4: Pump slices through the full range 2D-IR spectrum of reduced *Cr*HydA1 as shown in Fig. S2. The pump frequencies correspond to the fundamental transitions of the $H_{sred}H^+$ state. Signals labelled with an asterisk have significant contributions from other states. The pump slice at 1883 cm^{-1} has H_{ox} contributions and very weak bleach signals indicating coupling to several CN modes, including those of the $H_{sred}H^+$ state, expected at 2029 and 2070 cm^{-1}.

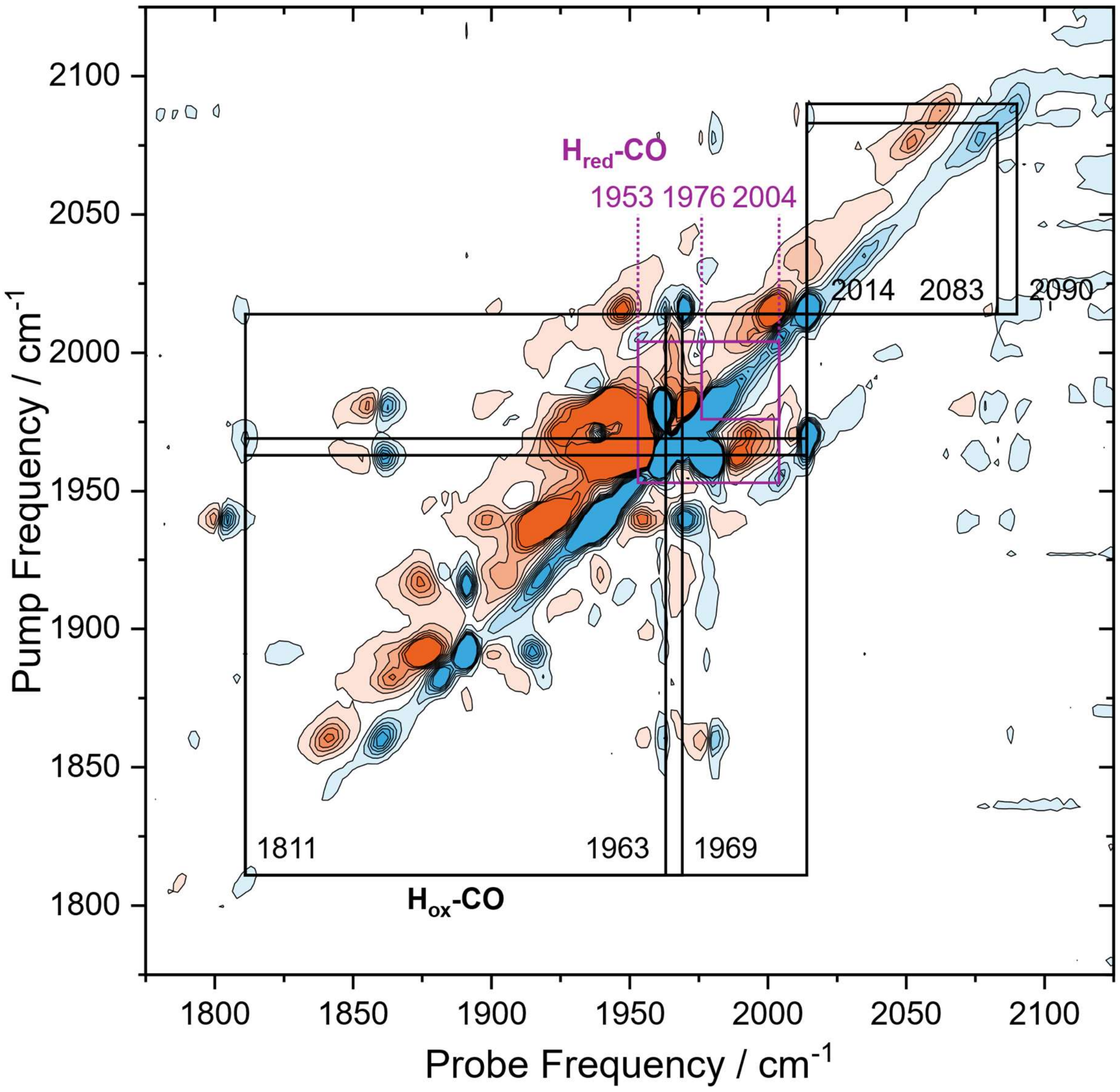

Figure S5: Full-range 2D-IR spectrum of reduced *Cr*HydA1 (Fig. 2 A), covering all CO and CN stretch-mode signals. The spectrum shows the same data as Fig. S2 but at different contour levels. Prominent signals related to H_{red}-CO and H_{ox}-CO are highlighted in purple and black, respectively. The spectrum was recorded with perpendicular pump-probe polarization at a waiting time of T_W = 250 fs at T = 283 K.

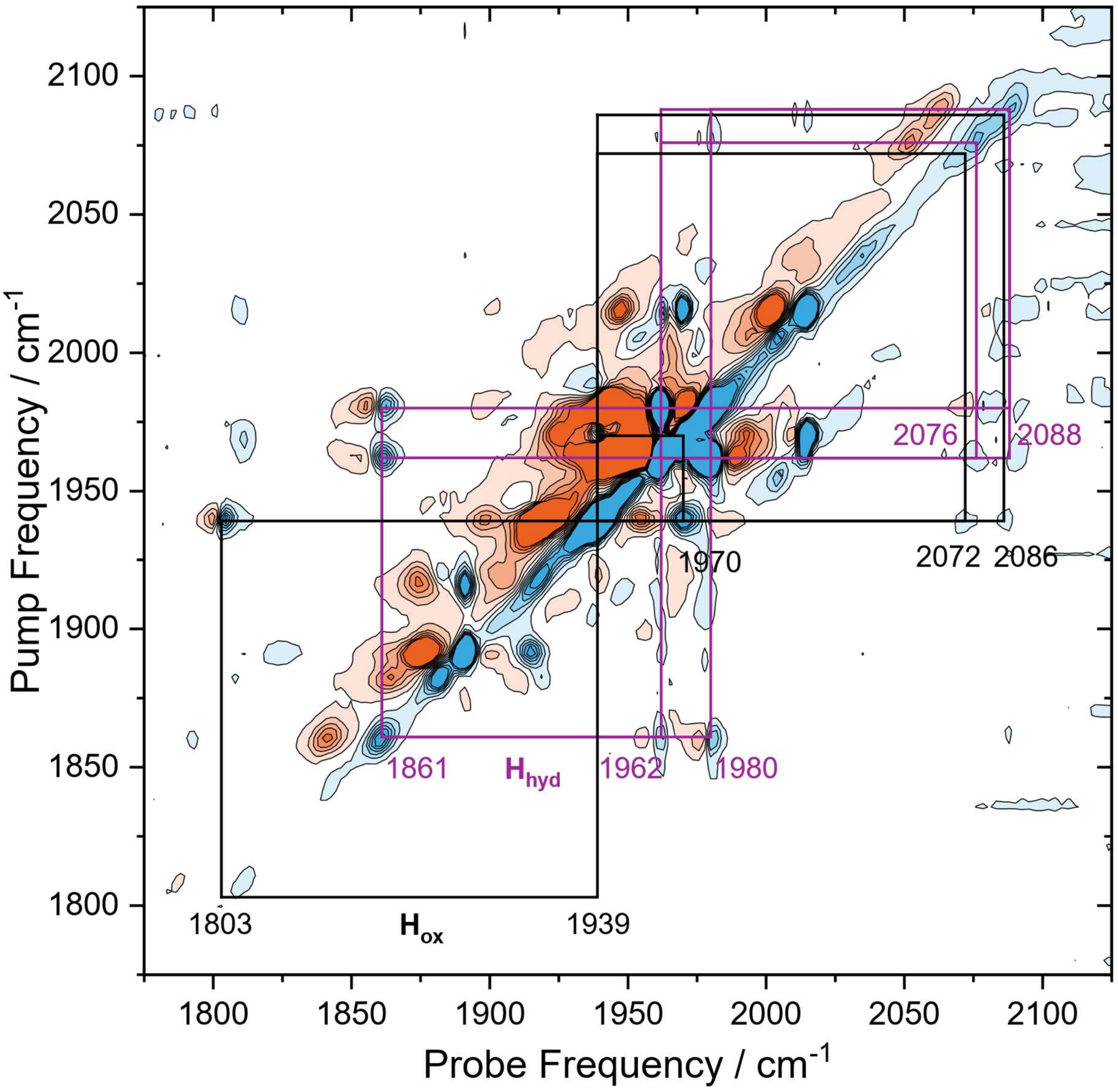

Figure S6 Full-range 2D-IR spectrum of reduced *Cr*HydA1 (Fig. 2A), covering all CO and CN stretch-mode signals. The spectrum shows the same data as Fig. S2 and Fig. S5. The contour levels are identical to Fig. S5. Prominent signals related to H_{hyd} and H_{ox} (or possible H_{red}'H, which has very similar IR bands) are highlighted in purple and black, respectively. The spectrum was recorded with perpendicular pump-probe polarization at a waiting time of T_W = 250 fs at T = 283 K.

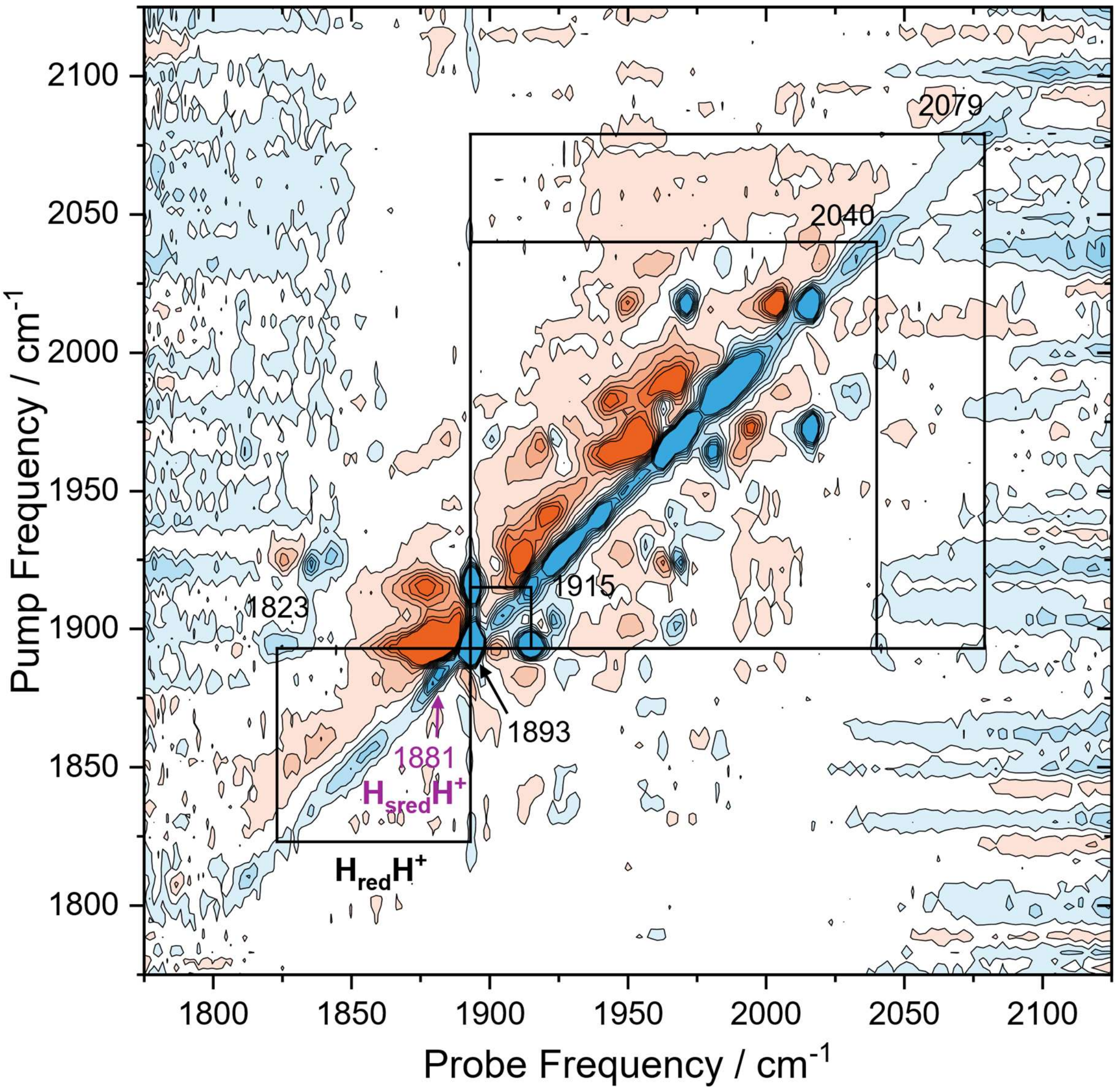

Figure S7: Full-range 2D-IR spectrum of reduced *Dd*HydAB (Fig. 2B), covering all CO and CN stretch-mode signals. Prominent signals related to $H_{red}H^+$ are highlighted in black with a potential contribution of the strongest $H_{sred}H^+$ state highlighted in purple. The spectrum was recorded with perpendicular pump-probe polarization at a waiting time of T_W = 250 fs at T = 283 K.

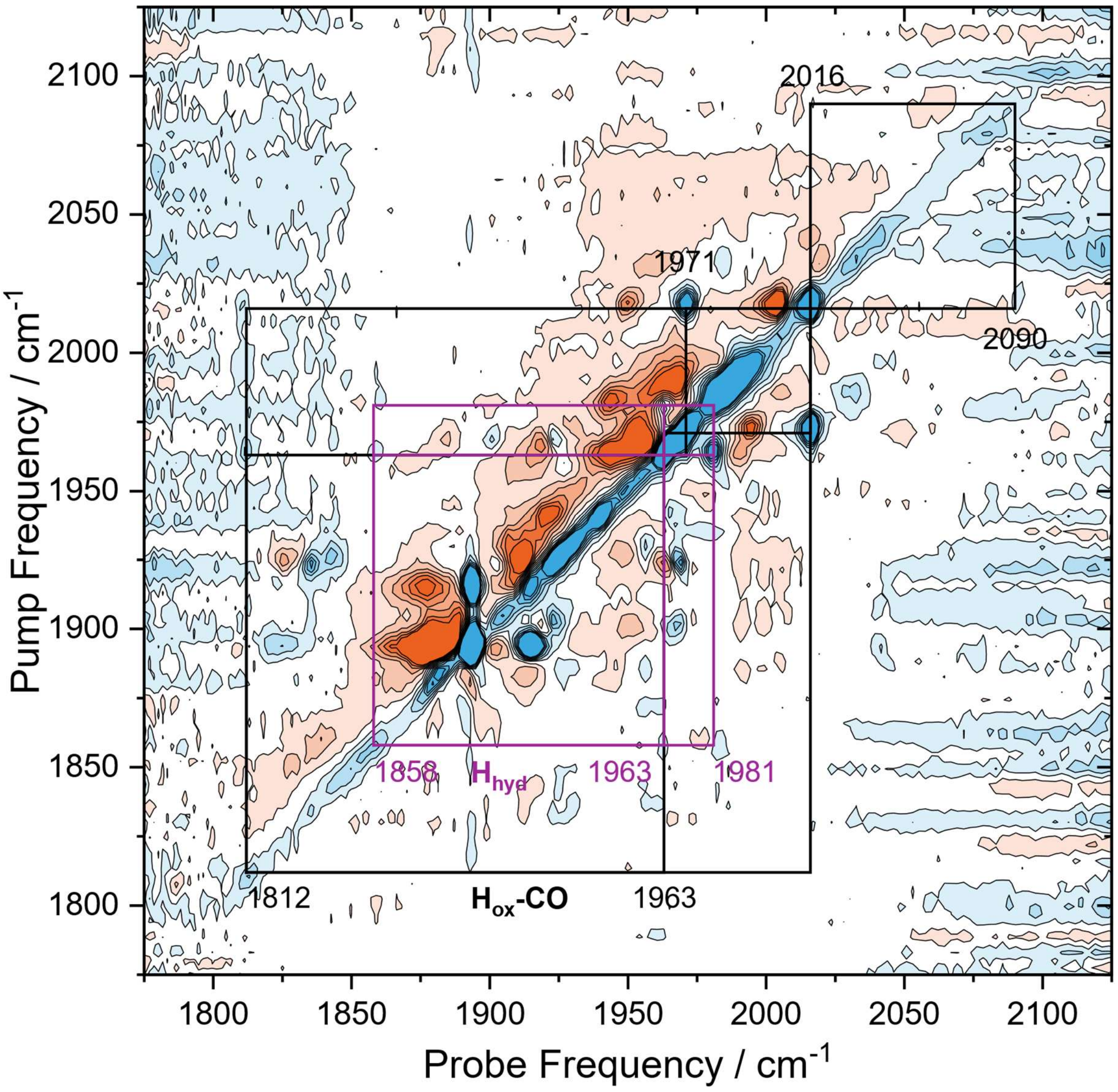

Figure S8: Full-range 2D-IR spectrum of reduced *Dd*HydAB (Fig. 2B), covering all CO and CN stretch-mode signals. The spectrum shows the same data as Fig. S7. Prominent signals related to H_{ox}-CO are highlighted in black, and the presence of some H_{hyd} contribution is highlighted in purple. The spectrum was recorded with perpendicular pump-probe polarization at a waiting time of T_W = 250 fs at T = 283 K.

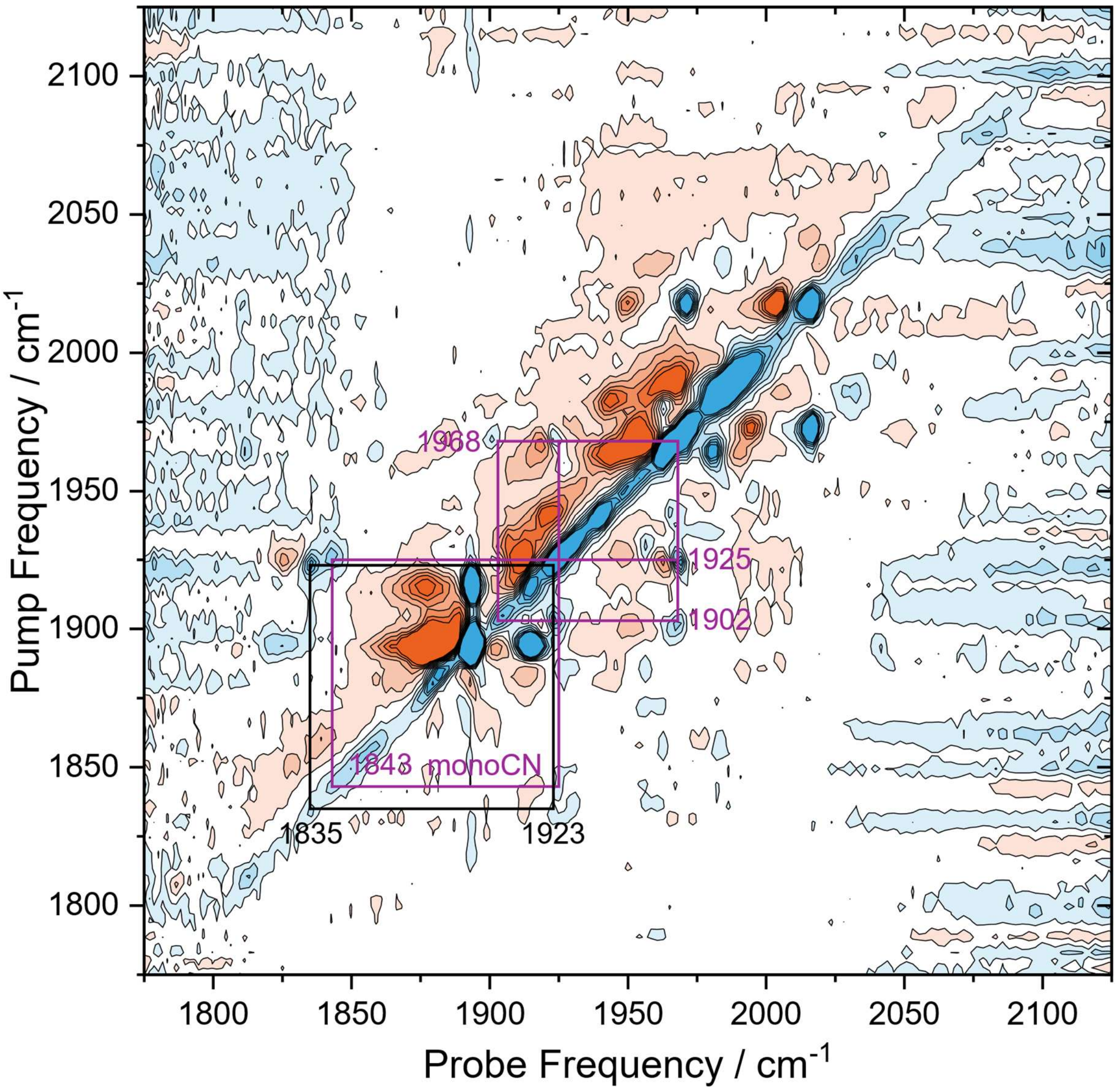

Figure S9: Full-range 2D-IR spectrum of reduced *Dd*HydAB (Fig. 2B), covering all CO and CN stretch-mode signals. The spectrum shows the same data as Fig. S7 and Fig. S8. Prominent signals of a small fraction of enzyme that has been maturated by a mono-CN version of the $[2Fe]^{ADT}$ cofactor are highlighted in purple. This is a contaminant due to the artificial maturation process for *Dd*HydAB.[1, 7] Black refers to a possible, previously unidentified, sub-state of the monoCN enzyme. The spectrum was recorded with perpendicular pump-probe polarization at a waiting time of T_W = 250 fs at T = 283 K.

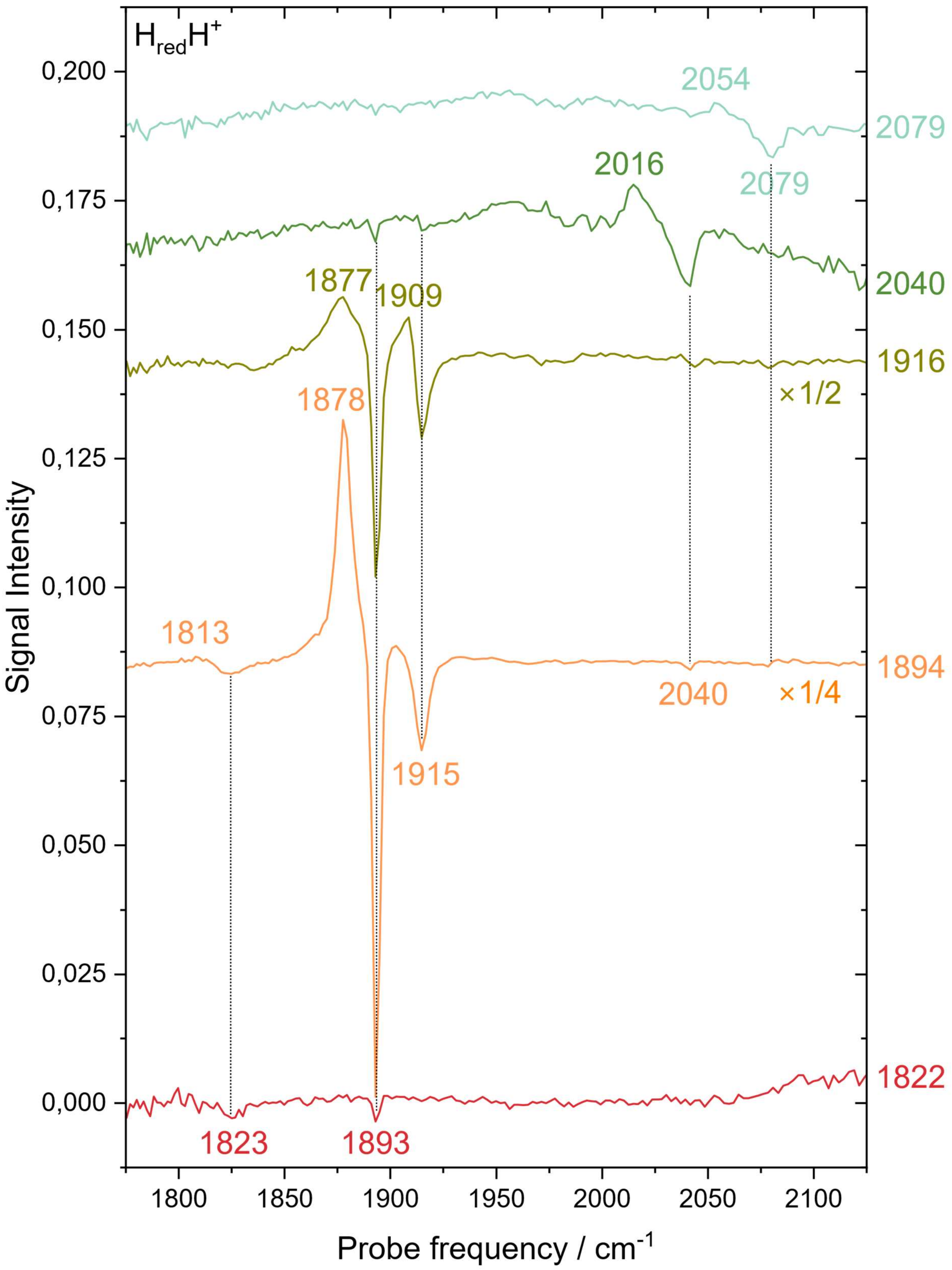

Figure S10: Pump slices through the full range 2D-IR spectrum of reduced *Dd*HydAB as shown in Fig. S7. The pump frequencies correspond to the fundamental transitions of the $H_{red}H^+$ state.

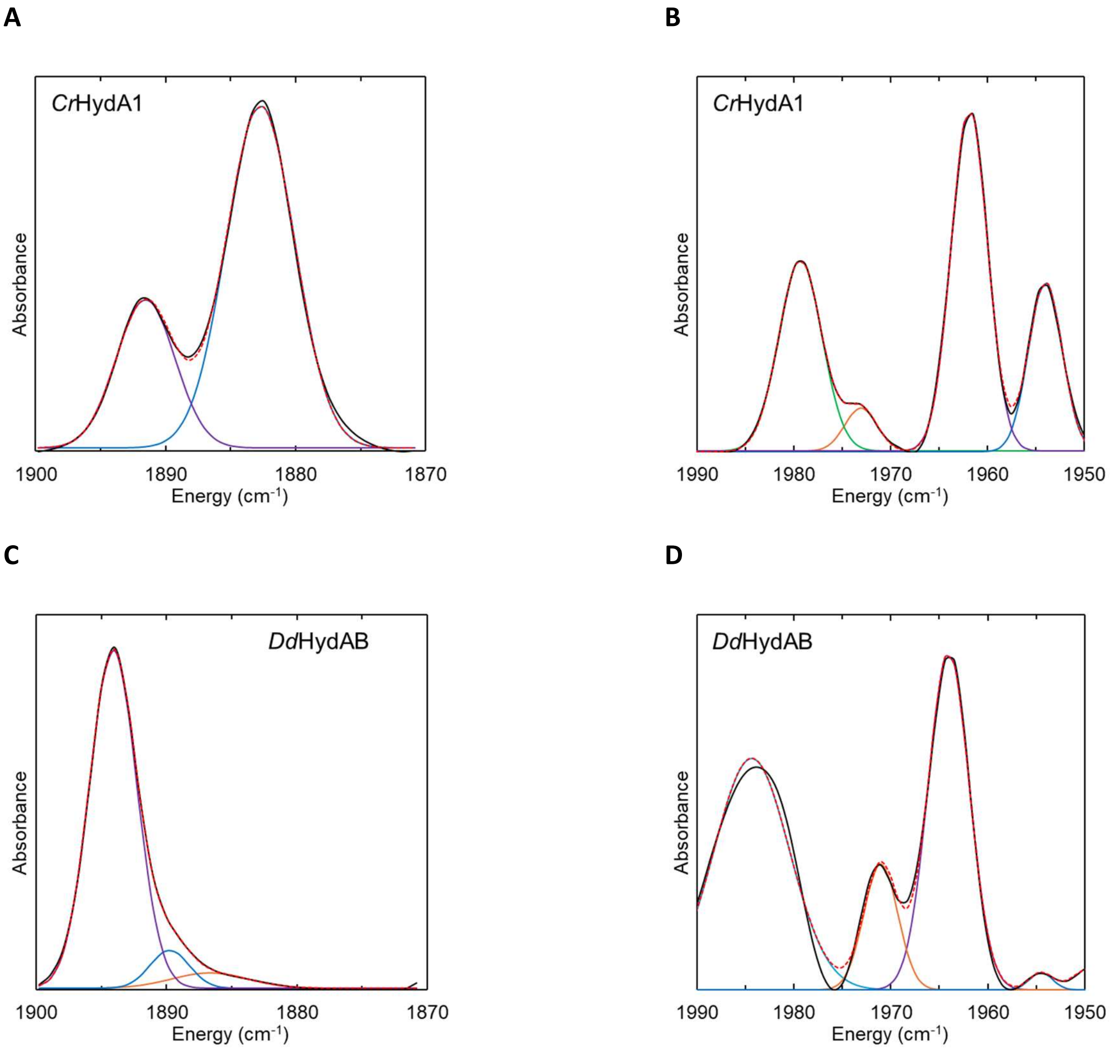

Figure S11: Attempts at fitting the linear IR spectra from samples of *Cr*HydA1 poised predominantly in the $H_{sred}H^+$ state (**A/B**) and *Dd*HydAB poised predominantly in the $H_{red}H^+$ state (**C/D**), fitted with Gaussian peaks in the regions between 1870 cm^{-1} and 1900 cm^{-1} (**A/C**), where the most intense peaks from the $H_{red}H^+$ and $H_{sred}H^+$ states are found) and 1950 cm^{-1} and 1990 cm^{-1} (**B/D**) (where the where the most intense peaks from the $H_{hyd:ox}$ and $H_{hyd:red}$ states are found). While the region from 1870 cm^{-1} to 1900 cm^{-1} clearly shows a terminal CO peak associated with $H_{red}H^+$ (1892 cm^{-1} for *Cr*HydA1 and 1894 cm^{-1} for *Dd*HydAB) and a terminal CO peak associated with $H_{sred}H^+$ (1882 cm^{-1} for *Cr*HydA1 and 1884 cm^{-1} for *Dd*HydAB – note an additional species at 1889 cm^{-1}), the region between 1950 cm^{-1} and 1990 cm^{-1} is complicated as it contains several overlapping peaks. Moreover, the observed peak at 1954 cm^{-1} for *Cr*HydA1 is known to contain peaks from both $H_{hyd:ox}$ and $H_{hyd:red}$ and the distinct peaks for $H_{hyd:ox}$ (1983 cm^{-1}) and $H_{hyd:red}$ (1972 cm^{-1}) overlap with features from the H_{ox}-CO state (1970 cm^{-1}) and another form of the H_{hyd} state that normally accumulates at low pH in the presence of sodium dithionite (1980 cm^{-1}). The same region for *Dd*HydAB contains a very weak feature at 1954 cm^{-1} (probably from a tiny amount of the $H_{hyd:red}$ state in tautomeric equilibrium with a very small amount of $H_{sred}H^+$), intense peaks at 1964 cm^{-1} and 1971 cm^{-1} (which may be partially from $H_{hyd:ox}$, but overlap with the 1963 cm^{-1} and 1972 cm^{-1} peaks from H_{ox}-CO), and an intense broad feature at 1984 cm^{-1} that was previously attributed to *Dd*HydAB with exogenously bound CN^- from the artificial maturation process. We do not know for certain that the most intense peaks in the $H_{red}H^+$, $H_{sred}H^+$, $H_{hyd:ox}$ and $H_{hyd:red}$ states have the same extinction coefficients and peak areas, but if we assume this is the case then we can obtain ratios of of $H_{sred}H^+/H_{hyd:red}$ of 7.6 for *Cr*HydA1 and 6.4 for *Dd*HydAB, and ratios of of $H_{sred}H^+/H_{hyd:red}$ of 1.3 for *Cr*HydA1 and 2.5 for *Dd*HydAB. This yields the ΔG values of +1.2 and +1.1 kcal/mol for the formation

for $H_{hyd:red}$ from $H_{sred}H^+$ in *Cr*HydA1 and *Dd*HydAB, respectively, and +0.16 and +0.54 kcal/mol for the formation for $H_{hyd:ox}$ from $H_{sred}H^+$ in *Cr*HydA1 and *Dd*HydAB, respectively. Overall, this indicated that the hydride tautomers are slightly less stable than the reduced/protonated forms but not by much.

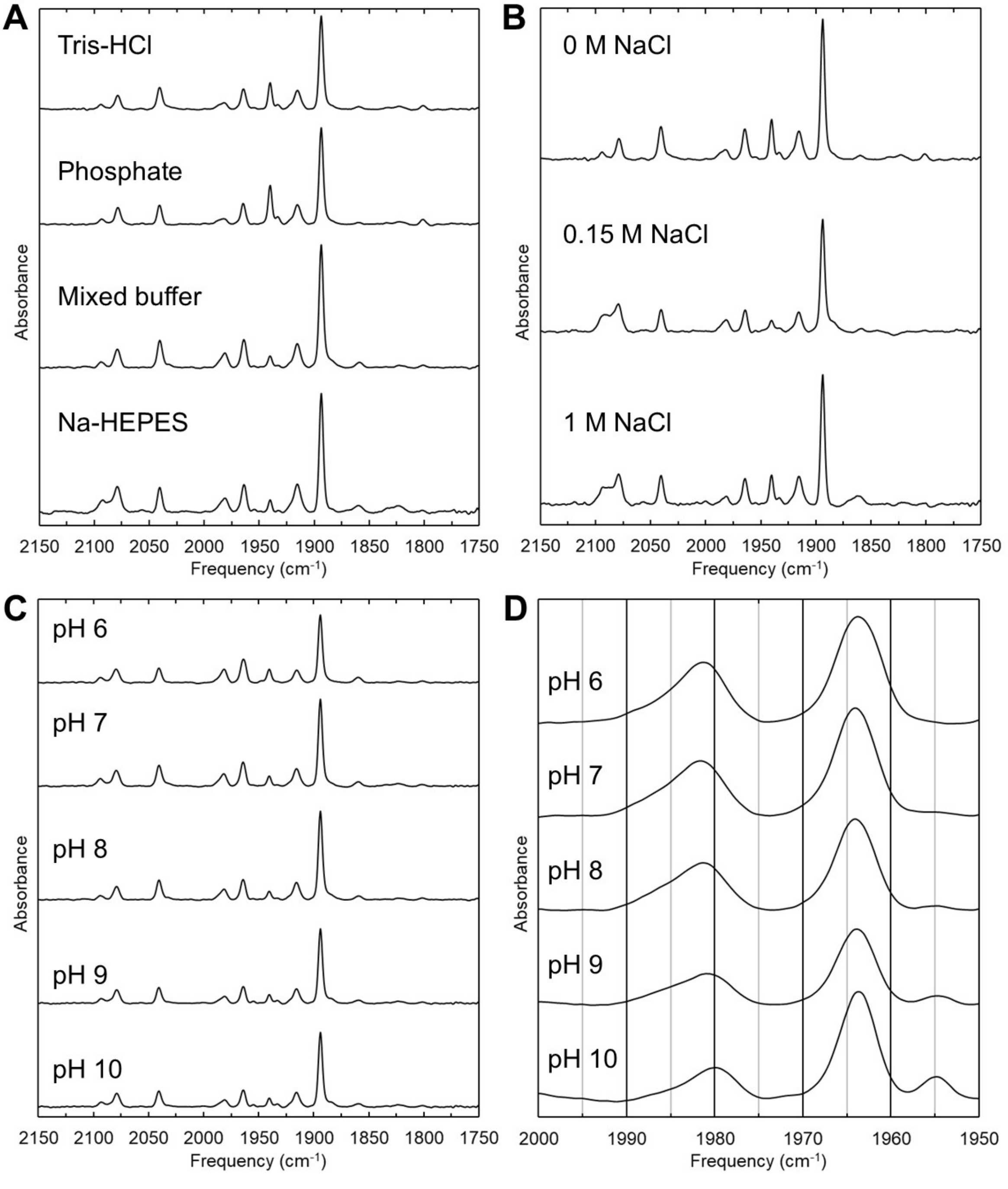

Figure S12: Samples of *Dd*HydAB were exchanged into various buffers and reduced with 20 mM sodium dithionite to obtain predominantly the $H_{red}H^+$ state. A) Comparison of samples at pH 8 in 100 mM Tris-HCl, 100 mM sodium phosphate and 100 mM Na-HEPES, all containing no additional NaCl, and 100 mM mixed buffer (15 mM sodium acetate, 15 mM Na-MES, 15 mM Na-HEPES, 15 mM Na-TAPS and 15 mM Na-CHES), containing 100 mM NaCl. B) Comparison of samples in 100 mM Tris-HCl containing 0 M, 0.15 M or 1 M NaCl. C) Comparison of samples in 100 mM mixed buffer pH corrected to 6, 7, 8, 9 or 10, containing 100 mM NaCl. D) Expansion of the region from C between 1950 cm^{-1} and 2000 cm^{-1}.

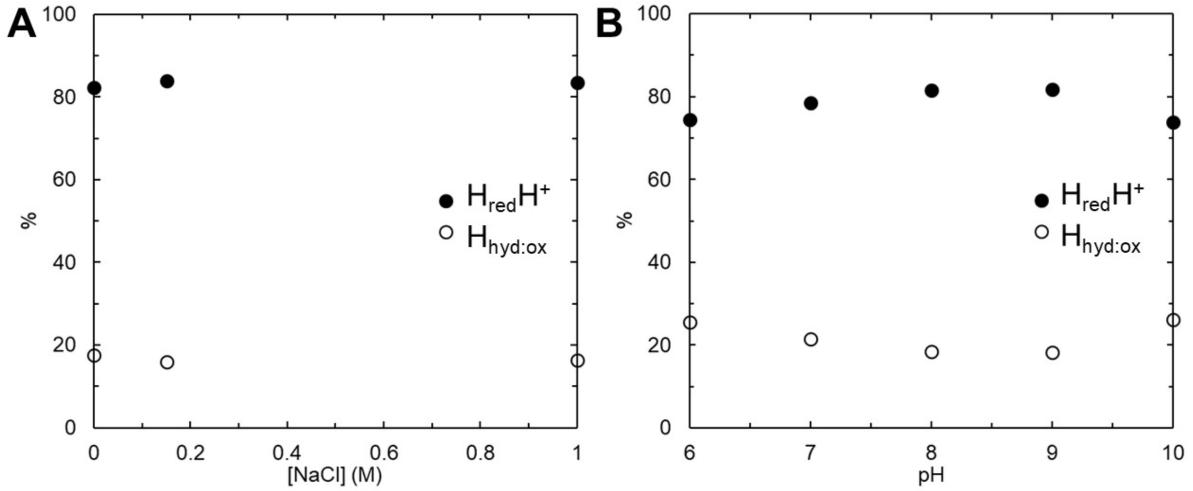

Figure S13: Plots of the variation in % fraction of $H_{red}H^+$ (filled circles) and $H_{hyd:ox}$ (empty circles) states with A) NaCl concentration and B) pH. Data are those from Table S2.

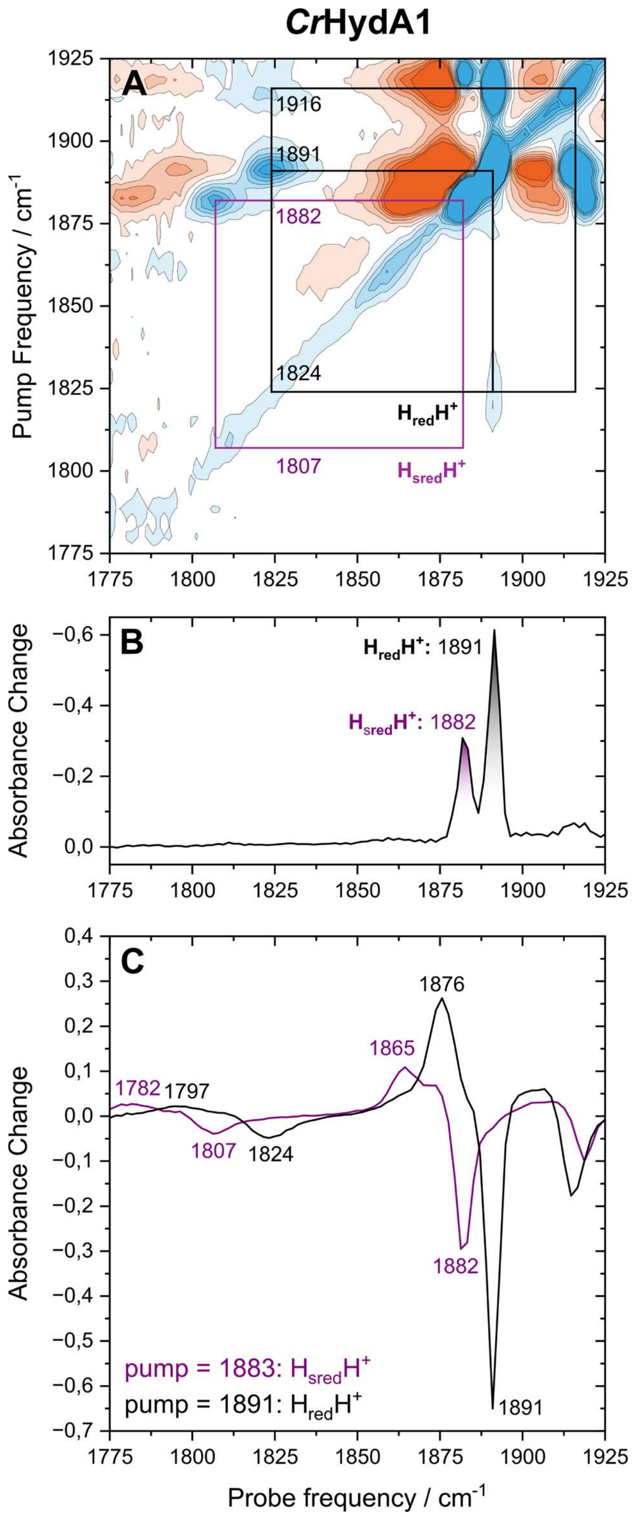

Figure S14: (A) 2D-IR spectrum of *Cr*HydA1, reduced with sodium dithionite at pH 8. (B) Sign-inverted diagonal spectrum. (C) Pump slices through the 2D-IR spectrum. Cross-peaks between the most intense tCO mode and the µCO mode are highlighted for $H_{red}H^+$ (black) and $H_{sred}H^+$ (purple). Spectra were recorded with perpendicular pump-probe polarisation at T = 283 K and T_W = 3 ps.

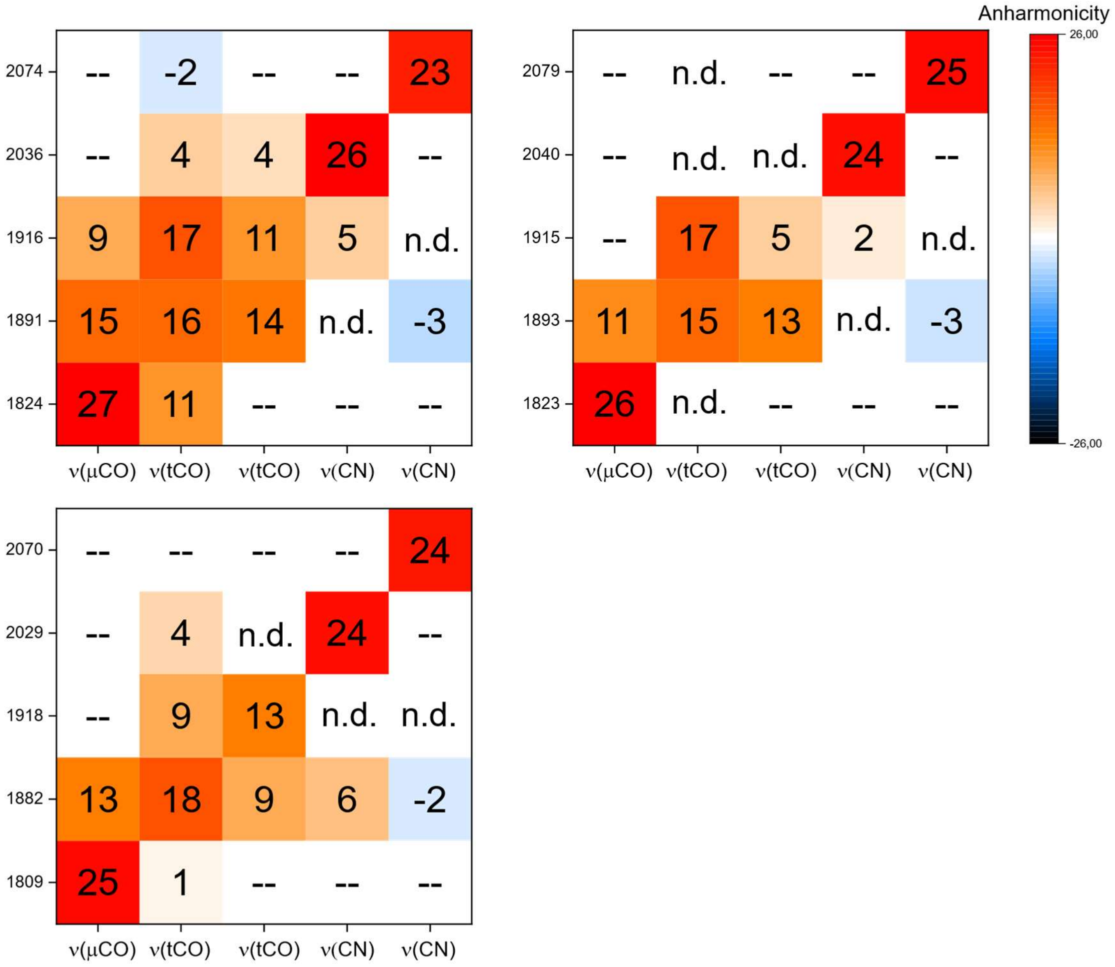

Figure S15: Overview of apparent anharmonicities of the main species discussed in the manuscript. Top left: $H_{red}H^+$ of *Cr*HydA1. Top right: $H_{red}H^+$ of *Dd*HydAB. Bottom left: $H_{sred}H^+$ of *Cr*HydA1. Values were calculated from peak positions derived by fitting Gaussian line shapes to the experimental data. The absence of observable cross peaks is indicated by "--", while "n.d." indicates cross peaks for which the anharmonicity could not be determined, due to a low intensity of the excited-state-absorption signal. The diagonal anharmonicities of the µCO mode are extracted from the energy-transfer cross peaks of the 3 ps measurement (see Figure 3) and have a substantially higher fit error of ca. +/- 5 cm^{-1}.

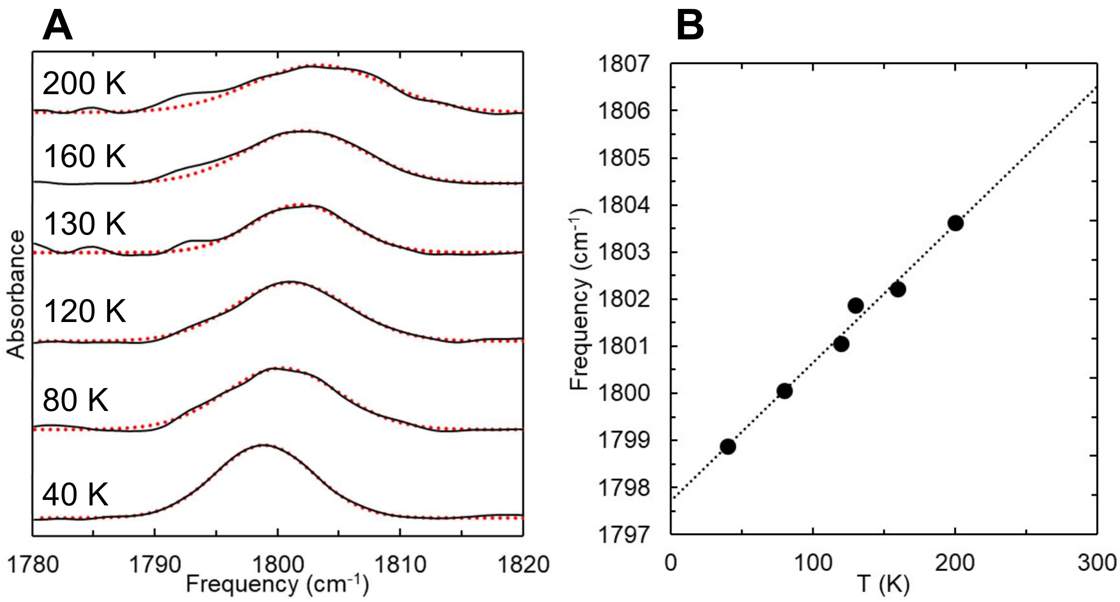

Figure S16: Analysis of the bridging CO peak position in cryo infrared spectroscopy of the $H_{sred}H^+$ state from *Cr*HydA1. Data were collected previously.[8] (A) A Gaussian function was fitted to the µCO peak using QSOAS [9] for datasets collected between 40 K and 200 K. Above 200 K the peak intensity was too low to give robust fits, while at lower temperatures the temperature could not be set accurately. (B) The peak position was plotted as a function of temperature and fitted with a linear curve. Datapoints above the protein glass transition point (ca. 220 K) are not following the same trend and are therefore excluded from the fit.

Supplementary Tables

Table S1: Fundamental frequencies (in cm^{-1}) for all CO and CN stretching modes of $H_{red}H^+$ and $H_{sred}H^+$, as observed in 2D-IR spectra recorded from *Cr*HydA1 and *Dd*HydAB. Note that $H_{sred}H^+$ was hardly populated for *Dd*HydAB, so that fundamental frequencies could not be determined for most modes.

Enzyme	State	µCO	tCO	tCO	tCN	tCN
*Cr*HydA1	$H_{red}H^+$	1824	1891	1915	2036	2074
	$H_{sred}H^+$	1809	1882	1918	2029	2070
*Dd*HydAB	$H_{red}H^+$	1823	1893	1915	2040	2079
	$H_{sred}H^+$	-	1881	-	-	-

Table S2: Absolute IR absorbance of the most intense peaks at 1894 cm^{-1} and 1963 cm^{-1} for the $H_{red}H^+$ and $H_{hyd:ox}$ states in *Dd*HydAB in various buffers in the presence of 20 mM sodium dithionite (values obtained from the data presented in Figure S12). % fraction of the two states were calculated assuming the same extinction coefficient for each state.

	Abs. $H_{red}H^+$ (1894 cm^{-1})	Abs. $H_{hyd:ox}$ (1964 cm^{-1})	% $H_{red}H^+$	% $H_{hyd:ox}$
100 mM HEPES, pH 8, 0 M NaCl	0.0041	0.0009	81.5	18.5
100 mM sodium phosphate, pH 8, 0 M NaCl	0.0033	0.0007	82.9	17.1
100 mM Tris-HCl, pH 8, 0 M NaCl	0.0032	0.0007	82.4	17.6
100 mM Tris-HCl, pH 8, 0.15 M NaCl	0.0026	0.0005	84.0	16.0
100 mM Tris-HCl, pH 8, 1 M NaCl	0.0030	0.0006	83.6	16.4
100 mM mixed buffer, pH 6, 0 M NaCl	0.0032	0.0011	74.5	25.5
100 mM mixed buffer, pH 7, 0 M NaCl	0.0042	0.0011	78.5	21.5
100 mM mixed buffer, pH 8, 0 M NaCl	0.0043	0.0010	81.6	18.4
100 mM mixed buffer, pH 9, 0 M NaCl	0.0036	0.0008	81.8	18.2
100 mM mixed buffer, pH 10, 0 M NaCl	0.0031	0.0011	73.8	26.2

Supplementary References

[1] J. A. Birrell, K. Wrede, K. Pawlak, P. Rodriguez-Maciá, O. Rüdiger, E. J. Reijerse, W. Lubitz, Artificial Maturation of the Highly Active Heterodimeric [FeFe] Hydrogenase from Desulfovibrio desulfuricans ATCC 7757. *Israel Journal of Chemistry* **2016**, *56*, 852-863.

[2] J. Esselborn, C. Lambertz, A. Adamska-Venkatesh, T. Simmons, G. Berggren, J. Noth, J. Siebel, A. Hemschemeier, V. Artero, E. Reijerse, M. Fontecave, W. Lubitz, T. Happe, Spontaneous activation of [FeFe]-hydrogenases by an inorganic [2Fe] active site mimic. *Nature Chemical Biology* **2013**, *9*, 607-609.

[3] L. P. DeFlores, R. A. Nicodemus, A. Tokmakoff, Two-dimensional Fourier transform spectroscopy in the pump-probe geometry. *Opt. Lett.* **2007**, *32*, 2966-2968.

[4] S.-H. Shim, D. B. Strasfeld, Y. L. Ling, M. T. Zanni, Automated 2D IR spectroscopy using a mid-IR pulse shaper and application of this technology to the human islet amyloid polypeptide. *Proceedings of the National Academy of Sciences* **2007**, *104*, 14197-14202.

[5] S.-H. Shim, M. T. Zanni, How to turn your pump–probe instrument into a multidimensional spectrometer: 2D IR and Vis spectroscopiesvia pulse shaping. *Physical Chemistry Chemical Physics* **2009**, *11*, 748-761.

[6] G. M. Greetham, P. Burgos, Q. Cao, I. P. Clark, P. S. Codd, R. C. Farrow, M. W. George, M. Kogimtzis, P. Matousek, A. W. Parker, M. R. Pollard, D. A. Robinson, Z.-J. Xin, M. Towrie, Ultra: A Unique Instrument for Time-Resolved Spectroscopy. *Applied Spectroscopy* **2010**, *64*, 1311-1319.

[7] M. Lorenzi, J. Gellett, A. Zamader, M. Senger, Z. Duan, P. Rodríguez-Maciá, G. Berggren, Investigating the role of the strong field ligands in [FeFe] hydrogenase: spectroscopic and functional characterization of a semi-synthetic mono-cyanide active site. *Chemical Science* **2022**, *13*, 11058-11064.

[8] J. A. Birrell, V. Pelmenschikov, N. Mishra, H. Wang, Y. Yoda, K. Tamasaku, T. B. Rauchfuss, S. P. Cramer, W. Lubitz, S. DeBeer, Spectroscopic and Computational Evidence that [FeFe] Hydrogenases Operate Exclusively with CO-Bridged Intermediates. *Journal of the American Chemical Society* **2020**, *142*, 222-232.

[9] V. Fourmond, QSoas: A Versatile Software for Data Analysis. *Analytical Chemistry* **2016**, *88*, 5050-5052.
